# STAT1-L351F is associated with enhanced interferon signaling and susceptibility to *Talaromyces marneffei* infection

**DOI:** 10.3389/fimmu.2026.1813775

**Published:** 2026-04-20

**Authors:** Yuchen Gu, Haimei Zhang, Qiaozi Lin, Guoguo Ye, Dongli Lian, Xinru Li, Yingxia Liu, Jing Yuan, Liping Guo

**Affiliations:** 1 Shenzhen Key Laboratory of Pathogen and Immunity, National Clinical Research Center for Infectious Disease, State Key Discipline of Infectious Disease, Shenzhen Third People's Hospital, Second Hospital Affiliated to Southern University of Science and Technology, Shenzhen, China; 2 School of Medicine, Southern University of Science and Technology, Shenzhen, China; 3 School of Public Health, Shenzhen University Medical School, Shenzhen, China; 4 Department of Infectious Diseases, The First Affiliated Hospital of Guangxi Medical University, Nanning, China; 5 State Key Laboratory of Respiratory Disease, National Clinical Research Center for Respiratory Disease, National Center for Respiratory Medicine, Guangzhou Institute of Respiratory Health, The First Affiliated Hospital of Guangzhou Medical University, Guangzhou, Guangdong, China; 6 Hargen Biotechnology (Shenzhen) Co., Ltd, Shenzhen, China; 7 Key Laboratory of Basic Research on Regional Diseases, Guangxi Medical University, Education Department of Guangxi Zhuang Autonomous Region, Nanning, China

**Keywords:** IL-17, interferon, MDSC, NKT cells, ruxolitinib, STAT1 gain-of-function, *Talaromyces marneffei*, talaromycosis

## Abstract

**Introduction:**

Talaromyces marneffei can cause life-threatening disseminated infection, yet the immune alterations linking STAT1 gain-of-function (GOF) variants, including the previously reported L351F site, to talaromycosis remain incompletely defined.

**Methods:**

We investigated STAT1-L351F in a four-member pedigree in which disease was restricted to the proband, combining human immunophenotyping, mechanistic assays, and an in vivo infection model. Systemic immune status was assessed by serum cytokine profiling, and peripheral blood mononuclear cell (PBMC) antifungal control was quantified using CFU-based ex vivo growth assays with T. marneffei, with extension to Cryptococcus neoformans and Candida albicans. STAT1 signaling was evaluated in HEK293T cells expressing STAT1-WT or STAT1-L351F by measuring IFN-α/β/γ-induced STAT1 phosphorylation and interferon-stimulated response element/gamma-activated sequence (ISRE/GAS) reporter activity. Wild-type and Stat1-L351F knock-in mice were challenged intranasally with T. marneffei and analyzed for lung fungal burden, histopathology, immune profiling, and bulk lung transcriptomics; infected Stat1-L351F mice additionally received ruxolitinib to probe the impact of JAK inhibition on infection-associated myeloid responses.

**Results:**

The proband displayed an interferon-skewed cytokine milieu, and patient PBMCs supported increased ex vivo growth of T. marneffei and C. neoformans, whereas C. albicans did not show a consistent increase. STAT1-L351F was associated with increased IFN-induced STAT1 phosphorylation and enhanced ISRE/GAS reporter activity, consistent with a hyper-responsive interferon signaling state in this system. In vivo, female Stat1-L351F mice developed higher lung fungal burden and more severe pathology at 16 days post-infection, accompanied by reduced NKT-cell frequencies and broad transcriptomic remodeling characterized by type I interferon-biased signatures together with dysregulated myeloid/neutrophil effector and IL-17-related programs. Ruxolitinib partially attenuated infection-associated expansion of myeloid-derived suppressor cell subsets.

**Discussion:**

Overall, STAT1-L351F was associated with impaired antifungal control, selective ex vivo permissiveness to distinct fungal pathogens, and altered IL-17-linked immune programs during T. marneffei infection, providing a multi-layered framework for understanding how dysregulated STAT1 signaling may reshape antifungal immunity across human, cell-based, and in vivo systems.

## 1 Introduction

*Talaromyces marneffei* is a dimorphic fungal pathogen capable of causing disseminated infection and severe morbidity, particularly in individuals with immunodeficiency or inborn errors of immunity (1–6). Despite advances in diagnostics (including Mp1p antigen detection and metagenomic next-generation sequencing), host factors that govern susceptibility and immunopathology remain incompletely defined, and mechanistic insight is needed to guide risk stratification and potential adjunctive immunomodulatory approaches (7–11).

STAT1 is a central transcription factor downstream of type I and type II interferons (IFN-I and IFN-II), coordinating antimicrobial defense through canonical JAK–STAT signaling (12, 13) Heterozygous STAT1 gain-of-function (GOF) variants are associated with broad immune dysregulation and susceptibility to fungal infections (14–18). While IFN hyper-responsiveness is a consistent molecular hallmark of STAT1-GOF, mutation-specific transcriptional fingerprints and diverse interferon-signature gene responses have been reported (19–21). How exaggerated interferon signaling translates into impaired antifungal protection, particularly against *T. marneffei*, remains unresolved (14, 17).

Recent clinical observations increasingly suggest that severe or recurrent *T. marneffei* infection may be linked to underlying immunodeficiency, including inborn errors of immunity. Such cases provide a “natural experiment” to illuminate host pathways required for effective antifungal control. In this context, dysregulated STAT1–interferon signaling has emerged as a biologically plausible contributor, yet a key mechanistic gap remains: it is still unclear why STAT1 gain-of-function (GOF), despite heightened interferon responsiveness, predisposes to invasive fungal disease. Addressing this question requires an integrated evidence chain spanning human immunophenotyping and ex vivo infection assays, mechanistic cell-based signaling readouts, and *in vivo* infection models.

Protective antifungal immunity requires integrated innate and adaptive programs, including phagocyte-mediated containment and T-cell-dependent pathways such as Th17/IL-17 responses (22, 23). Pattern-recognition receptor pathways such as Dectin-1 can shape antifungal inflammation and interferon programs, highlighting the tight coupling between fungal sensing and downstream cytokine networks (22, 24). These observations raise the possibility that STAT1-GOF-driven interferon bias disrupts key antifungal effector networks, including IL-17-associated axes and innate-like lymphoid contributions (23, 25).

Clarifying how STAT1-GOF-driven interferon bias disrupts antifungal effector networks is clinically important for risk stratification and monitoring of invasive fungal disease in STAT1-GOF, and may inform rational adjunctive immunomodulatory strategies. Here, we investigated a STAT1-L351F variant, previously reported within the spectrum of STAT1 gain-of-function mutations, using an integrated human-to-mechanism framework including (i) family genetic and immune profiling, (ii) ex vivo PBMC fungal growth assays (with *T. marneffei* as the primary pathogen and *C. neoformans*/*C. albicans* as extension comparators), (iii) cell-based STAT1 signaling readouts, and (iv) an intranasal *T. marneffei* infection model in female STAT1-L351F knock-in mice with transcriptomic and immune profiling. We additionally included proof-of-concept pathway modulation using the JAK1/2 inhibitor ruxolitinib to explore whether infection-associated immune remodeling can be partially reshaped *in vivo*.

## 2 Methods

### 2.1 Genetic analysis

Peripheral blood samples were obtained from a patient with recurrent *Talaromyces marneffei* infection. Genomic DNA was extracted from peripheral blood leukocytes and subjected to whole-exome sequencing (WES), which identified a heterozygous STAT1 c.G1053T (p.L351F) variant in exon 12. The variant was subsequently confirmed by Sanger sequencing. Primer sequences and PCR conditions are provided in the Supplementary Methods.

### 2.2 Serum cytokine measurement and PBMC immunoblotting

Peripheral blood was obtained from the index patient during a single clinical sampling and from healthy donors. Serum was isolated by centrifugation and stored at −80 °C until analysis. Serum concentrations of IFN-α, IFN-β, IFN-γ, IL-27, IL-12, IL-23, IL-22, and IL-6 were quantified using a commercially available cytokine detection kit (Xinbosheng, China) according to the manufacturer’s instructions. Cytokine concentrations were calculated based on standard curves generated for each analyte. In parallel, peripheral blood mononuclear cells (PBMCs) were isolated by density-gradient centrifugation using Ficoll-Paque (GE Healthcare). PBMCs from the patient and healthy donors were lysed immediately after isolation without ex vivo cytokine stimulation, and protein extracts were subjected to immunoblotting to assess STAT1 pathway activation. Immunoblotting was performed using antibodies against total STAT1, phospho-STAT1, STAT2, phospho-STAT2, TYK2, phospho-TYK2, IFNAR1, IFNAR2, IRF9, and GAPDH as a loading control. Experiments involving patient-derived PBMCs were repeated in independent assay runs, and representative immunoblots are shown.

### 2.3 PBMC isolation and *ex vivo* fungal growth assays

Freshly isolated PBMCs from the patient and healthy donors were used immediately and resuspended in complete RPMI 1640 medium supplemented with 10% fetal bovine serum (FBS) and plated at 1 × 10^6^ cells per well in a final volume of 1 mL. PBMCs were separately co-cultured with *Talaromyces marneffei*, *Cryptococcus neoformans*, or *Candida albicans* at a final inoculum of 1 × 10^6^ CFU/mL for each fungal species. For *T. marneffei*, yeast-phase organisms were used to better mimic the *in vivo* pathogenic form. After 72 h of incubation at 37 °C with 5% CO_2_, whole cultures (cells plus supernatant) were thoroughly resuspended to capture both cell-associated and extracellular organisms. Host cells were disrupted to release cell-associated fungi, and suspensions were serially diluted and plated onto Potato Dextrose Agar (PDA) plates, incubated at 37 °C for 4 days, and colonies were counted for colony-forming unit (CFU) enumeration. Fungal growth was quantified as relative fold CFU (outgrowth), normalized to the mean CFU counts obtained from healthy donor PBMCs processed in parallel within the same experimental run. Specifically, the endpoint CFU for each sample was divided by the mean endpoint CFU of healthy donor PBMCs from the same experiment to determine the relative fold increase in fungal growth. Definition of n: patient samples were available from a limited number of individuals; therefore, n denotes independent experimental runs performed on different days using separately prepared aliquots and parallel healthy donor controls. Yeast-phase *T. marneffei* was generated by culturing in Potato Dextrose Broth (PDB) at 37 °C for 24 h prior to co-culture.

### 2.4 HEK293T cell culture, transfection, and STAT1 signaling assays

HEK293T cells and STAT1-knockout (STAT1-KO) HEK293T cells were maintained in DMEM supplemented with 10% fetal bovine serum (FBS) and 1% penicillin–streptomycin at 37 °C with 5% CO_2_. STAT1-KO HEK293T cells were generated using CRISPR/Cas9-mediated genome editing. Cells were transiently transfected with empty vector, STAT1 wild-type (STAT1-WT), or STAT1-L351F expression plasmids using Lipofectamine 3000 (Thermo Fisher Scientific) according to the manufacturer’s instructions. Equal amounts of plasmid DNA were used for transfection in all conditions. For luciferase reporter assays, cells were co-transfected with ISRE- or GAS-driven firefly luciferase reporter plasmids together with a Renilla luciferase plasmid as an internal control. At 24 h post-transfection, cells were stimulated with recombinant human IFN-α, IFN-β, or IFN-γ (100 ng/mL) for 18 h. Firefly luciferase activity was normalized to Renilla luciferase activity and expressed as fold induction relative to unstimulated controls. In parallel, STAT1 phosphorylation was assessed by immunoblotting following IFN-α stimulation (18 h). All experiments were performed at least three times independently.

### 2.5 Animals, *T. marneffei* infection, and ruxolitinib treatment

Female wild-type (WT) and Stat1-L351F knock-in mice (6–8 weeks old) were housed under specific pathogen-free (SPF) conditions. For the infection model, mice were challenged intranasally with *T. marneffei* (1 × 10^5 CFU in a total volume of 50 μL per mouse) on day 0, followed by a secondary challenge on day 7. To evaluate the effect of JAK inhibition, a cohort of infected WT and Stat1-L351F mice received ruxolitinib (RUX; 90 mg/kg) via oral gavage once daily from day 7 to day 16 post-infection (dpi). Body weights were monitored daily, and all mice were euthanized at 16 dpi for downstream tissue analysis. Experimental groups comprised WT-TM, 351-TM, WT-TM+RUX, and 351-TM+RUX.

### 2.6 Evaluation of lung fungal burden and histopathology

At 16 days post-infection (16 dpi), mice were euthanized and lungs were harvested and weighed. For fungal burden quantification, lung tissues were homogenized, serially diluted, and plated onto potato dextrose agar (PDA). After incubation at 37 °C for 4 days, fungal colonies were enumerated and expressed as colony-forming units per gram of lung tissue (CFU/g). For histopathological assessment at the same time point, lung tissues were fixed in 10% neutral-buffered formalin, paraffin-embedded, and sectioned for hematoxylin and eosin (H&E) and periodic acid–Schiff (PAS) staining. Semi-quantitative pathology scoring was performed on three randomly selected fields (×200 magnification) per mouse, evaluating alveolar septal thickening, edema, hemorrhage, and inflammatory cell infiltration. Each parameter was graded on a 0–4 scale (0, absent; 1, mild; 2, moderate; 3, marked; 4, severe), and scores were averaged to obtain the final pathology score for each mouse.

### 2.7 Splenic immune profiling and flow cytometry

Single-cell suspensions were prepared from harvested spleens by mechanical dissociation and passed through a 200-mesh filter, followed by red blood cell lysis. For multi-parameter flow cytometry, 1 × 10^6^ viable cells were first identified using Zombie UV™ Fixable Viability Dye. Surface staining was performed with fluorochrome-conjugated antibodies (**Supplementary Table 1**). For analysis of innate and myeloid populations, splenocytes were stained with antibodies against CD45, CD3, CD49b, CD11b, Ly6G, Ly6C, and F4/80. NKT cells were defined as CD45^+^CD3^+^CD49b^+^ cells. Macrophages were identified as CD45^+^CD11b^+^F4/80^+^ cells. Myeloid-derived suppressor cells (MDSCs) were defined as CD45^+^CD11b+Gr-1^+^ cells, with monocytic MDSCs (M-MDSCs) defined as CD11b^+^Ly6G^-^Ly6C^high^ and granulocytic MDSCs (G-MDSCs) defined as CD11b^+^Ly6G^+^Ly6C^low^. After staining, cells were washed with PBS and resuspended in 300–500 µL PBS for acquisition on a CytoFLEX LX (Beckman Coulter) flow cytometer. At least 50,000 events were collected within the CD45^+^ viable gate. Flow cytometry data were analyzed using FlowJo software. Data are presented as frequencies of total CD45^+^ cells and summarized as mean ± SEM. Fluorescence intensity axes were displayed on logarithmic scales. Gating thresholds were initially determined using fluorescence-minus-one (FMO) controls and then applied consistently across all samples during analysis. Gating strategies are shown in **Supplementary Figure 1**.

### 2.8 Bulk lung RNA-seq and enrichment analysis

Total RNA was extracted from lung tissues collected at 16 dpi using TRIzol reagent (Invitrogen) according to the manufacturer’s instructions. RNA sequencing libraries were prepared using the NEBNext Ultra II RNA Library Prep Kit for Illumina and sequenced on an Illumina NovaSeq 6000 platform to generate paired-end reads. Clean reads were aligned to the mouse reference genome (GRCm38/mm10) using HISAT2. Differential gene expression between WT-TM and 351-TM female lung samples was analyzed using the DESeq2 package in R, with false discovery rate (FDR) correction applied for multiple testing. Genes with an adjusted P value < 0.05 were considered differentially expressed. Gene Ontology (GO) and Kyoto Encyclopedia of Genes and Genomes (KEGG) pathway enrichment analyses were performed using the clusterProfiler package in R, and adjusted P values were used to determine significance. Data visualization was conducted using R software. Additional targeted gene set enrichment analysis (GSEA) was performed on the full ranked differential-expression profile (351-TM vs WT-TM) using preranked gene set enrichment analysis. Genes were ranked by the DESeq2 Wald statistic, and mouse Hallmark, GO biological process, and Reactome gene sets were used to evaluate reviewer-relevant inflammatory and antimicrobial effector modules, including interferon responses, myeloid/leukocyte activation, neutrophil chemotaxis, phagocytosis, respiratory burst, degranulation, and ROS-related pathways. Positive normalized enrichment scores (NES) indicate relative enrichment in 351-TM, whereas negative NES indicate relative enrichment in WT-TM.

### 2.9 Use of AI-assisted technologies

During the preparation of this work, the authors used ChatGPT/Gemini in order to improve the readability and language fluency of the manuscript. After using this tool, the authors reviewed and edited the content as needed and took full responsibility for the content of the publication.

### 2.10 Statistics

Data are shown as mean ± SEM unless otherwise specified. Two-group comparisons were analyzed using unpaired two-tailed t tests with Welch’s correction. Comparisons involving more than two groups were analyzed by one-way or two-way ANOVA as appropriate, with multiple-comparison correction (Sidak’s or Tukey’s). Body weight curves were analyzed using two-way ANOVA with multiple comparisons. Statistical significance was defined as * (P < 0.05), ** (P < 0.01), *** (P < 0.001) and ****(P < 0.0001).

## 3 Results

### 3.1 Identification of STAT1-L351F and an interferon-skewed immune phenotype in a family with fungal susceptibility

**Figure 1:**
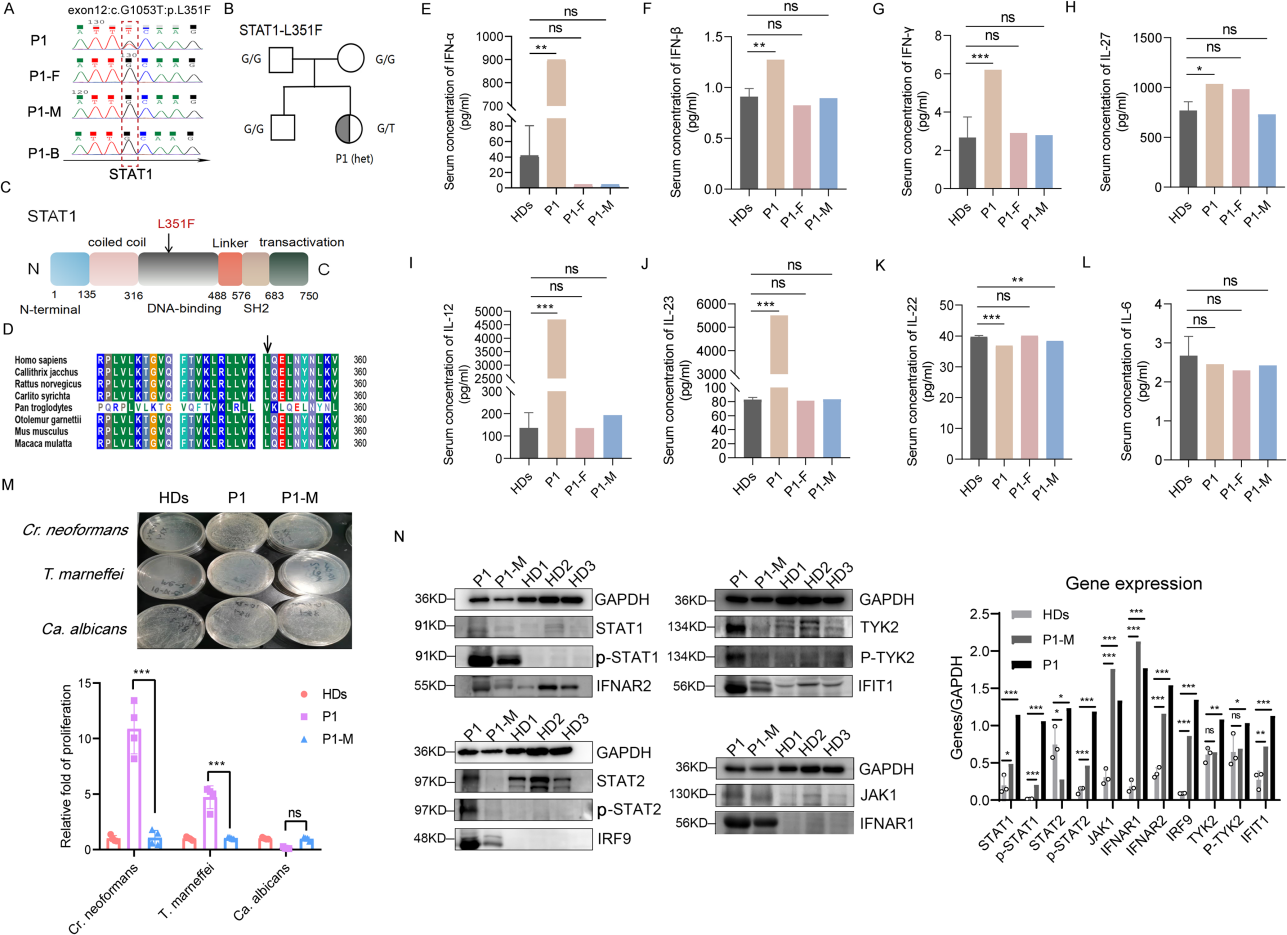
STAT1-L351F is associated with an interferon-skewed immune phenotype and increased ex vivo fungal outgrowth. **(A)** Sanger sequencing identifying STAT1 exon 12 c.G1053T (p.L351F). **(B)** Pedigree of the family. **(C)** STAT1 domain schematic showing the position of L351F in the DNA-binding domain. **(D)** Conservation of L351 across species. **(E–L)** Serum cytokine concentrations as indicated. **(M)** Representative plates and quantification of fungal outgrowth in PBMC cultures following ex vivo infection with *C. neoformans*, *T. marneffei*, or *C. albicans*, expressed as relative fold change normalized to healthy donor samples processed in parallel. **(N)** Immunoblotting analysis of STAT1 signaling and interferon pathway components in unstimulated PBMC lysates. Healthy donor (HD) samples represent independent biological donors. Measurements from the index patient were obtained from a single blood collection and repeated in independent assay runs.

In the index patient with recurrent *T. marneffei* infection, a heterozygous STAT1 exon 12 c.G1053T (p.L351F) variant was identified by whole-exome sequencing and confirmed by Sanger sequencing (**Figure 1A**). Pedigree analysis demonstrated that the variant was absent in both parents and the healthy sibling, indicating a *de novo* mutation (**Figure 1B**). The L351F substitution localizes to the STAT1 DNA-binding domain (**Figure 1C**) and affects a highly conserved residue across species (**Figure 1D**). Serum cytokine profiling revealed a pronounced interferon-skewed immune phenotype in the index patient. Compared with healthy donors (HDs), serum concentrations of IFN-α, IFN-β, and IFN-γ were markedly elevated (**Figures 1E–G**), whereas unaffected family members showed cytokine levels comparable to controls. Serum IL-27 was also increased in the index patient (**Figure 1H**), while IL-6 levels did not show consistent elevation (**Figure 1L**). Additional cytokine analyses demonstrated selective perturbation of interferon-associated and Th17-related pathways. Serum IL-12 and IL-23 levels were elevated in the index patient (**Figures 1I, J**), accompanied by a modest increase in IL-22 (**Figure 1K**), changes that were not observed in unaffected family members.

### 3.2 STAT1-L351F patient PBMCs show increased ex vivo fungal outgrowth and elevated STAT1 signaling

Ex vivo PBMC infection/co-culture assays revealed a selective defect in antifungal control associated with the STAT1-L351F variant. Compared with healthy donors processed in parallel, PBMCs from the index patient supported significantly increased ex vivo fungal outgrowth of *Talaromyces marneffei*, as quantified by CFU enumeration from whole PBMC co-cultures (**Figure 1M**). A similar increase in fungal growth was observed for *Cryptococcus neoformans*, whereas *Candida albicans* did not exhibit enhanced proliferation under identical assay conditions. Notably, PBMCs from the unaffected family member (P1-M) showed fungal control comparable to that of healthy donors, indicating that the observed phenotype was patient-specific. To interrogate the underlying signaling state, immunoblotting analyses of patient PBMCs demonstrated increased basal phosphorylation of STAT1, accompanied by enhanced activation of associated JAK–STAT pathway components, including TYK2 and STAT2, relative to healthy controls (**Figure 1N**). Consistent with these findings, protein levels of interferon pathway components, including IFNAR1, IFNAR2, and IRF9, were increased in patient PBMCs compared with controls (**Figure 1N**). Together, these results indicate that STAT1-L351F is associated with heightened interferon signaling in PBMCs concomitant with impaired control of select fungal pathogens.

### 3.3 STAT1-L351F is associated with increased interferon-induced STAT1 activation and transcriptional responses in HEK293T cells

**Figure 2:**
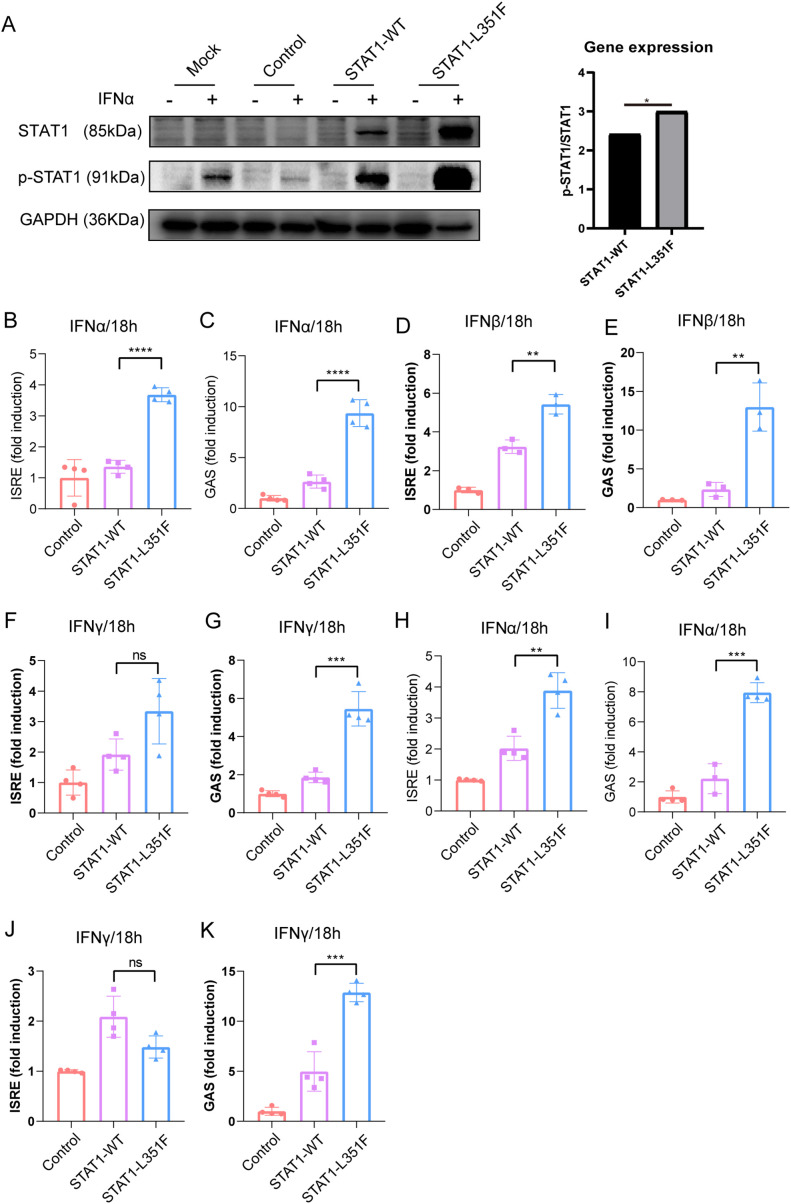
STAT1-L351F enhances STAT1 phosphorylation and IFN-responsive transcriptional activity in HEK293T cells. **(A)** Immunoblot analysis of total STAT1, phosphorylated STAT1 (p-STAT1), and GAPDH in HEK293T cells expressing empty vector (Control), STAT1-WT, or STAT1-L351F, with or without IFN-α stimulation for 18 h. Mock indicates cells without plasmid transfection. Equal amounts of plasmid DNA were used for transfection in all conditions. **(B–G)** ISRE- and GAS-driven dual-luciferase reporter activity in STAT1-sufficient HEK293T cells following stimulation with IFN-α, IFN-β or IFN-γ for 18 h. **(H–K)** ISRE- and GAS-driven dual-luciferase reporter activity in STAT1-knockout HEK293T cells reconstituted with STAT1-WT or STAT1-L351F following IFN-α or IFN-γ stimulation for 18 h. Data are presented as fold induction relative to unstimulated controls. All interferons were used at 100 ng/mL.

To directly assess the functional consequences of the STAT1-L351F variant, HEK293T cells were transfected with empty vector, STAT1-WT, or STAT1-L351F expression plasmids and stimulated with type I or type II interferons. Following IFN-α stimulation, immunoblotting analysis revealed increased STAT1 phosphorylation in cells expressing STAT1-L351F compared with STAT1-WT and vector controls (**Figure 2A**), consistent with increased STAT1 activation at the protein level. To evaluate downstream transcriptional responses, ISRE- and GAS-driven dual-luciferase reporter assays were performed in STAT1-sufficient HEK293T cells. Upon IFN-α stimulation, STAT1-L351F significantly enhanced both ISRE and GAS reporter activity relative to STAT1-WT (**Figures 2B, C**). IFN-β stimulation similarly resulted in increased ISRE and GAS activation in STAT1-L351F-expressing cells compared with controls (**Figures 2D, E**). In contrast, under IFN-γ stimulation, STAT1-L351F preferentially enhanced GAS reporter activity, whereas ISRE activation was not significantly increased (**Figures 2F, G**), consistent with the canonical signaling specificity of type II interferon.

To determine whether the observed transcriptional hyper-responsiveness depended on endogenous STAT1, parallel reporter assays were performed in STAT1-knockout HEK293T cells reconstituted with STAT1-WT or STAT1-L351F. In this background, STAT1-L351F expression led to significantly increased IFN-α–induced ISRE and GAS reporter activity compared with STAT1-WT (**Figures 2H, I**). Similarly, under IFN-γ stimulation, STAT1-L351F selectively enhanced GAS reporter activity without a significant increase in ISRE activation (**Figures 2J, K**). Together, these results show that STAT1-L351F is associated with increased interferon-induced STAT1 phosphorylation and enhanced ISRE/GAS reporter activity in this overexpression system.

### 3.4 STAT1-L351F knock-in mice exhibit increased lung fungal burden and pathology following intranasal *T. marneffei* infection

**Figure 3:**
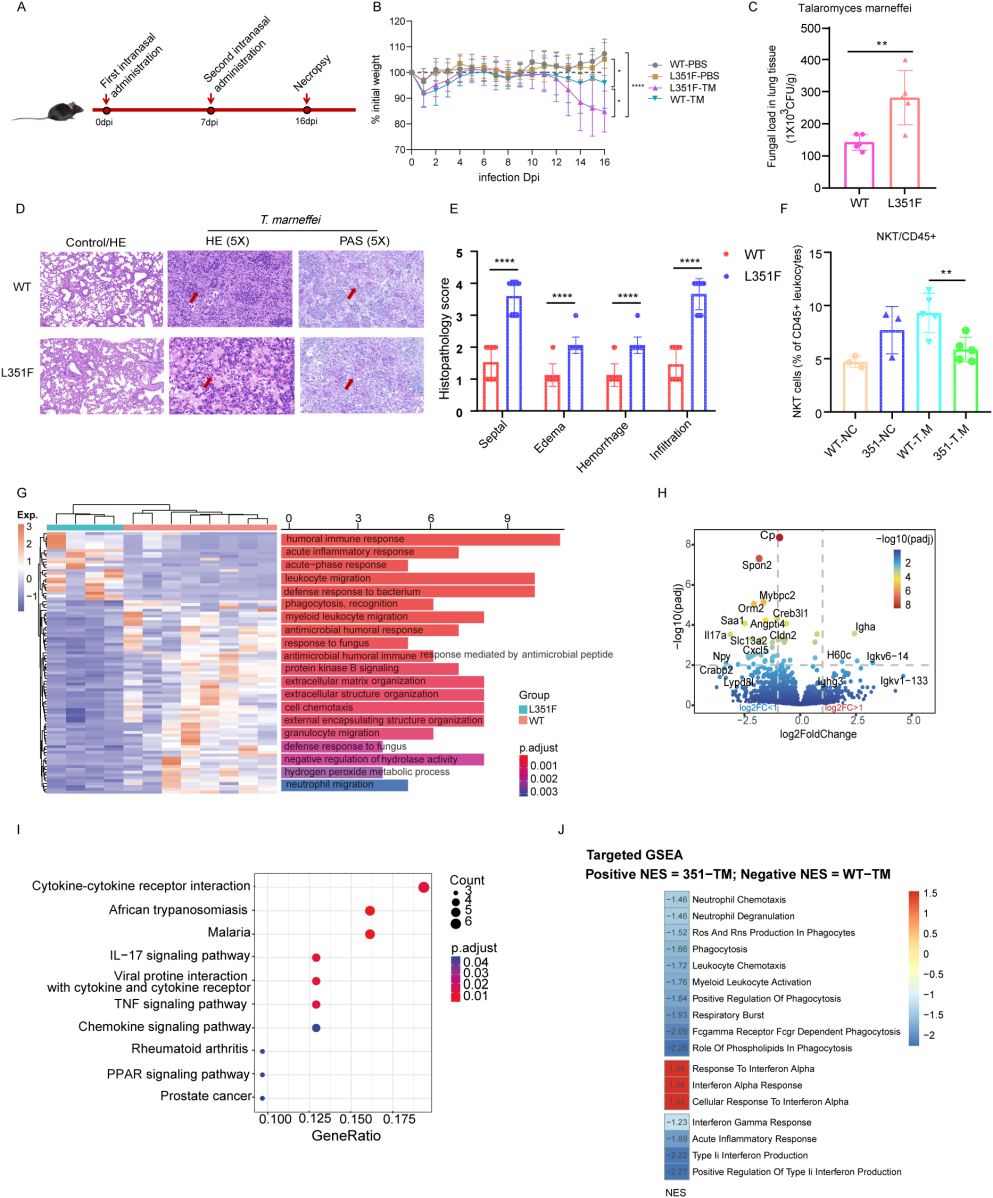
Female STAT1-L351F knock-in mice exhibit increased susceptibility to intranasal *T. marneffei* infection with immune remodeling and altered lung transcriptomics.**(A)** Experimental timeline for intranasal *T. marneffei* challenge in female mice (1 × 10^5 CFU in 50 μL, days 0 and 7; n=5 per group) with necropsy at 16 dpi. **(B)** Body weight changes during infection. **(C)** Lung fungal burden at 16 dpi (CFU/g). **(D)** Representative lung histology (H&E and PAS). **(E)** Semi-quantitative lung pathology scores at 16 dpi. See Methods for details of the scoring criteria. **(F)** Frequency of splenic NKT cells among CD45+ leukocytes at 16 dpi. **(G)** Heatmap of differentially expressed genes with Gene Ontology (biological process) enrichment (female lung bulk RNA-seq; 351-TM vs WT-TM). **(H)** Volcano plot of differentially expressed genes from the same comparison. **(I)** KEGG pathway enrichment analysis highlighting altered immune and inflammatory pathways, including IL-17 signaling. **(J)** Targeted preranked GSEA of reviewer-relevant inflammatory and antimicrobial effector modules. Positive NES indicates enrichment in 351-TM, whereas negative NES indicates enrichment in WT-TM. Type I interferon–associated signatures were relatively enriched in 351-TM lungs, whereas myeloid, neutrophil, phagocytic, respiratory burst, and type II interferon–related programs were preferentially enriched in WT-TM lungs.

Female WT and STAT1-L351F knock-in mice were subjected to a two-dose intranasal *T. marneffei* challenge (day 0 and day 7), with necropsy performed at 16 days post-infection (dpi) (**Figure 3A**). Body weight was monitored longitudinally throughout infection. Following challenge, STAT1-L351F mice exhibited greater infection-associated weight loss compared with WT controls, with a more pronounced and sustained decline over the course of infection (P< 0.05, **Figure 3B**). At the time of necropsy, STAT1-L351F mice displayed a significantly increased lung fungal burden compared with WT controls, as quantified by CFU per gram of lung tissue (**Figure 3C**), indicating impaired pulmonary control of *T. marneffei* infection in the presence of the STAT1 gain-of-function variant.

Histopathological examination of lung sections further demonstrated exacerbated tissue injury in STAT1-L351F mice. Representative H&E and PAS staining revealed more pronounced alveolar septal thickening, edema, hemorrhage, and inflammatory cell infiltration relative to WT-infected mice (**Figure 3D**). Semi-quantitative scoring of these four pathological features confirmed significantly higher composite pathology scores in STAT1-L351F lungs (P<0.0001, **Figure 3E**), consistent with more severe infection-associated lung damage.

### 3.5 Lung immune remodeling and transcriptomic alterations accompany *T. marneffei* infection in STAT1-L351F mice

To assess infection-associated immune remodeling, flow cytometric analysis of splenocytes was performed at 16 dpi. STAT1-L351F mice exhibited a significant reduction in NKT cells, expressed as a fraction of total CD45+ leukocytes, compared with WT-infected controls (**Figure 3F**), indicating selective alterations in splenic immune cell composition during infection.

To define broader molecular changes in the infected lung, bulk RNA sequencing was performed on lung tissues from female WT-TM and 351-TM mice. Differential gene expression analysis revealed extensive transcriptional remodeling in STAT1-L351F lungs. A heatmap of differentially expressed genes highlighted coordinated alterations in immune- and inflammation-related programs, accompanied by enrichment of Gene Ontology biological processes associated with cytokine signaling, leukocyte activation, and host defense responses (**Figure 3G**). The global distribution of transcriptional changes is summarized in a volcano plot, which revealed a predominance of downregulated genes in STAT1-L351F lungs compared with WT-TM controls (**Figure 3H**). Notably, several immune- and host defense–related genes were significantly reduced, including Cp, Spon2, IL17a, and Cxcl5, whereas a smaller subset of genes was upregulated, indicating an overall transcriptional suppression of select immune pathways in the lungs of STAT1-L351F mice during infection. Consistent with these observations, KEGG pathway enrichment analysis identified multiple immune and inflammatory pathways that were significantly altered in STAT1-L351F lungs, including cytokine–cytokine receptor interaction, TNF and chemokine signaling, and IL-17 related immune pathways (**Figure 3I**), highlighting widespread remodeling of lung immune programs associated with the STAT1-L351F variant during *T. marneffei* infection. To further refine the transcriptomic interpretation, we performed targeted preranked GSEA focused on reviewer-relevant inflammatory and antimicrobial effector modules. This reanalysis showed that type I interferon-associated signatures, including Interferon Alpha Response, Response to Interferon Alpha, and Cellular Response to Interferon Alpha, were relatively enriched in 351-TM lungs. In contrast, multiple pathways linked to coordinated antifungal defense were preferentially enriched in WT-TM, including Interferon Gamma Response, Acute Inflammatory Response, Type II Interferon Production, Myeloid Leukocyte Activation, Leukocyte/Neutrophil Chemotaxis, Phagocytosis, Respiratory Burst, Neutrophil Degranulation, and ROS/RNS Production in Phagocytes (**Figure 3J**). Together, these findings support a type I IFN-biased but functionally dysregulated immune remodeling pattern in STAT1-L351F lungs, rather than uniformly enhanced inflammatory host defense.

### 3.6 Ruxolitinib attenuates infection-associated MDSC changes in infected mice

**Figure 4:**
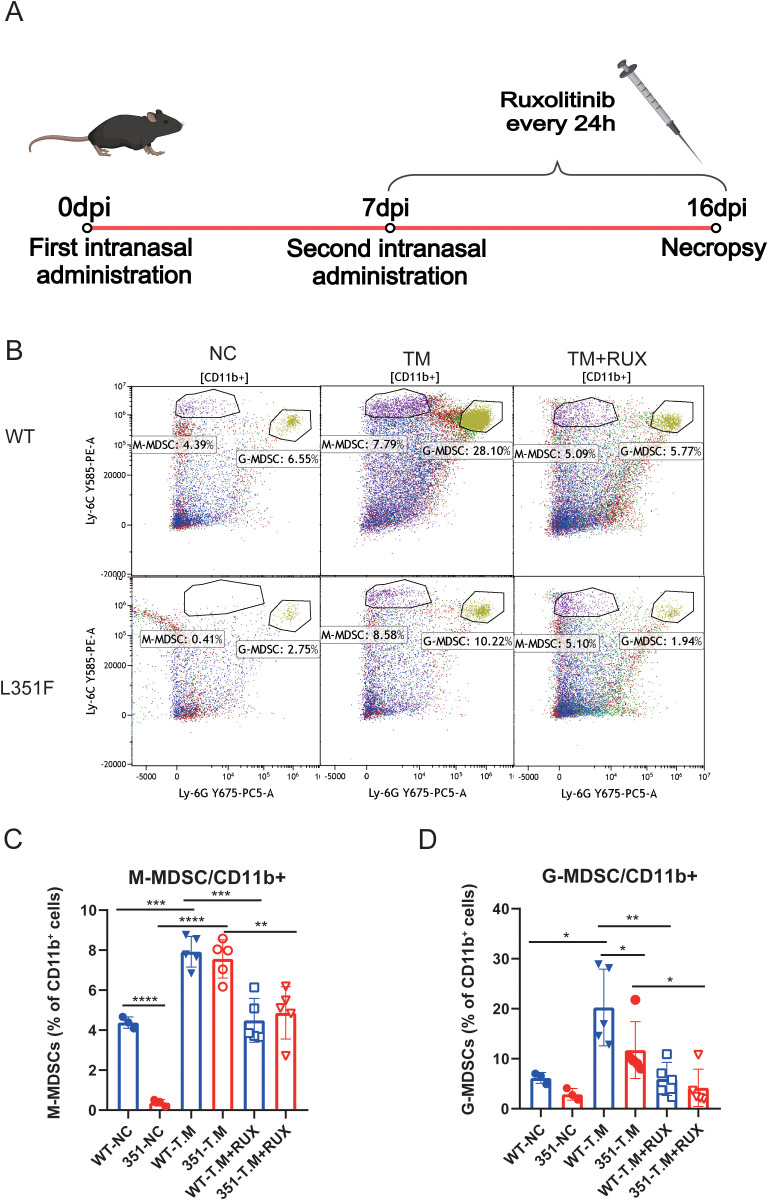
Ruxolitinib alters infection-associated splenic MDSC readouts in female mice. The gating strategy is provided in **Supplementary Figure 1**.**(A)** Infection and treatment timeline showing two intranasal *T. marneffei* administrations (Day 0 and Day 7) and daily ruxolitinib dosing every 24 h from Day 7 until necropsy at 16 dpi (90 mg/kg by oral gavage once every 24 h from Day 7 to Day 16). **(B)** Representative flow cytometry plots showing identification of M-MDSC (CD11b^+^Ly6G^-^Ly6C^high^) and G-MDSC (CD11b^+^Ly6G^+^Ly6C^low^) subsets within the CD11b^+^compartment from baseline, TM, and TM+RUX female spleens at 16 dpi. **(C)** Frequency of M-MDSCs among CD11b^+^ cells (M-MDSC/CD11b^+^, %). **(D)** Frequency of G-MDSCs among CD11b^+^ cells (G-MDSC/CD11b^+^, %). Groups shown include WT-TM, 351-TM, WT-TM+RUX, and 351-TM+RUX. Data are presented as mean ± SEM (n = 5 per group). Statistical analysis was performed using two-way ANOVA (genotype × treatment) followed by Sidak’s multiple comparisons test. *P < 0.05, **P < 0.01, ***P < 0.001, ****P < 0.0001.

To explore proof-of-concept pathway modulation, ruxolitinib was administered to infected female mice beginning at day 7 post-infection and continued daily until necropsy at 16 dpi (**Figure 4A**). Splenocytes were subsequently analyzed by flow cytometry to assess infection-associated myeloid remodeling. Flow cytometric gating within the CD11b^+^compartment revealed distinct populations of monocytic (M-MDSC; Ly6G^-^Ly6C^high^) and granulocytic (G-MDSC; Ly6G^+^Ly6C^low^) myeloid-derived suppressor cells. Representative gating plots from baseline, TM, and TM+RUX female spleens at 16 dpi illustrate the distribution of these subsets within the CD11b^+^compartment (**Figure 4B**). Quantitative analysis showed that STAT1-L351F mice exhibited markedly reduced baseline frequencies of M-MDSCs compared with WT controls, while *T. marneffei* infection induced substantial expansion of M-MDSCs in both genotypes, resulting in comparable levels in WT-TM and 351-TM groups (**Figure 4C**). Ruxolitinib treatment significantly reduced the frequency of M-MDSCs in both genotypes. Among infected mice, ruxolitinib reduced M-MDSC frequencies relative to genotype-matched untreated infected groups. Infection was also associated with increased G-MDSC frequencies, and ruxolitinib likewise reduced G-MDSC frequencies in infected WT and STAT1-L351F mice (**Figure 4D**). Together, these data indicate that pharmacologic JAK1/2 inhibition with ruxolitinib partially reshapes infection-associated MDSC expansion in the spleen, consistent with a potential contribution of JAK–STAT signaling to infection-associated myeloid remodeling. The detailed gating strategy for M-MDSC and G-MDSC identification is shown in **Supplementary Figure 1**.

## 4 Discussion

This study integrates human immunophenotyping, mechanistic STAT1 signaling assays, and a female-only knock-in infection model to investigate how STAT1-L351F is associated with susceptibility to *T. marneffei* infection. Across these layers, our data supports a unifying concept: exaggerated interferon-biased STAT1 signaling is associated with a reshaped antifungal immune landscape spanning circulating immune responses, tissue-level effector programs, and lung immunopathology during *T. marneffei* challenge (14, 19, 20).

At the human level, patient-derived findings pointed to an interferon-skewed inflammatory milieu together with increased ex vivo permissiveness of PBMCs to *T. marneffei* growth. Importantly, the ex vivo phenotype extended to *C. neoformans* but not *C. albicans*, suggesting that susceptibility associated with dysregulated STAT1 signaling may not be uniform across fungal species and may depend on pathogen-specific immune requirements and host–pathogen interaction niches. A plausible explanation is that these fungi rely on partially distinct protective immune modules. Both *T. marneffei* and *C. neoformans* are strongly shaped by interferon-dependent mononuclear phagocyte/macrophage immunity, whereas the best-established fungal phenotype in human STAT1 GOF is chronic mucocutaneous candidiasis, which is classically linked to impaired Th17/IL-17 immunity; moreover, host defense against invasive *C. albicans* infection depends heavily on neutrophil recruitment and fungicidal effector functions. Thus, the absence of increased *C. albicans* outgrowth in our assay should not be interpreted as preserved anti-Candida immunity in general, but rather as suggesting that STAT1-L351F may preferentially perturb IFN-dominant macrophage-associated antifungal programs that are particularly relevant to *T. marneffei* and *C. neoformans* under the conditions tested. Although limited by the number of donors, these observations align with the clinical impression that STAT1-GOF can predispose to invasive fungal disease and provide a functional readout that complements cytokine and signaling measurements (11, 14, 16–18). Because this PBMC outgrowth assay represents an integrated host-pathogen interaction readout, it does not distinguish specific antifungal effector mechanisms such as phagocytosis, intracellular killing, or oxidative responses.

Mechanistically, rather than establishing the GOF classification *de novo*, our HEK293T overexpression assays recapitulated an interferon-hyperresponsive signaling profile consistent with the previously reported gain-of-function behavior of STAT1-L351F. However, because intracellular transgene abundance was not directly quantified in the transient transfection experiments, these data should be interpreted as supportive functional evidence within this assay system rather than as a standalone mechanistic classification. This phenotype is characterized by increased STAT1 phosphorylation and amplified IFN-responsive ISRE/GAS transcriptional activity. Notably, the STAT1-L351F variant has recently been reported as a pathogenic STAT1 gain-of-function mutation in patients with immune dysregulation (26), supporting its classification within the STAT1-GOF spectrum. These data provide a tractable entry point linking genotype to pathway hyper-responsiveness. At the same time, heightened STAT1 activation should not be interpreted as broadly protective, particularly in the fungal setting; persistent interferon-biased signaling may instead reprogram downstream immune networks and alter the balance between protective effector responses and inflammatory pathology (12, 14, 19, 20). In this context, the key question is not whether interferon signaling is increased, but how this increase reshapes the coordination of antifungal immunity.

Our *in vivo* findings in female STAT1-L351F mice provide convergent evidence for increased susceptibility to intranasal *T. marneffei* infection. STAT1-L351F females displayed increased lung fungal burden at 16 dpi together with more severe tissue pathology, indicating that the STAT1 GOF state is accompanied by impaired fungal control and enhanced immunopathology at the site of infection. Splenic immune profiling further revealed reduced NKT cells among CD45+ leukocytes, suggesting that innate-like lymphoid compartments may be altered during infection in the STAT1 GOF setting. Consistent with these cellular changes, bulk lung transcriptomics demonstrated broad immune remodeling, with a type I interferon–biased but dysregulated antifungal transcriptional program, including IL-17 associated transcriptional programs (23, 25). While bulk RNA-seq cannot resolve the cellular sources of these signatures, the convergence of altered innate-like lymphocytes and transcriptomic immune pathway shifts supports a model in which STAT1 GOF disrupts the coordinated tissue programs required for effective antifungal control. Targeted GSEA further suggested a type I IFN-biased yet functionally uncoordinated immune state in STAT1-L351F lungs.

A possible mechanistic interpretation is that interferon-biased STAT1 signaling may be associated with altered coordination of protective antifungal networks, particularly those involving IL-17-associated axes and myeloid effector functions (23, 25). Interferon programs downstream of fungal sensing pathways (e.g., Dectin-1) can shape antifungal inflammation and cytokine networks, and our transcriptomic enrichment patterns are consistent with disruption of cytokine–cytokine receptor and IL-17-related programs during susceptibility (22–24). These findings motivate targeted follow-up at the cellular level to define whether Th17/γδ T cells/ILC3 compartments and phagocyte effector pathways are functionally impaired, and whether interferon-driven transcriptional states directly suppress antifungal effector functions or promote tissue-damaging inflammation. Clinically, these data support heightened vigilance for invasive fungal disease in STAT1-GOF and motivate mechanistically informed monitoring of interferon- and IL-17–linked immune states during infection (17).

Finally, we incorporated exploratory pathway modulation using the JAK1/2 inhibitor ruxolitinib as a proof-of-concept approach to test whether infection-associated immune remodeling can be modified *in vivo* (12, 27–31). In infected mice, ruxolitinib altered splenic MDSC readouts relative to genotype-matched untreated infected controls, consistent with an effect of JAK inhibition on myeloid immune states during infection. However, because WT-NC+RUX and 351-NC+RUX groups were not included, these data do not establish whether ruxolitinib corrects the steady-state myeloid phenotype associated with STAT1-L351F, nor do they distinguish selective reversal of GOF-associated dysregulation from a broader drug effect on myeloid compartments. Accordingly, the ruxolitinib findings should be interpreted as exploratory infected-state immune modulation rather than normalization of baseline immune homeostasis or evidence of therapeutic efficacy. Importantly, JAK inhibition can increase susceptibility to opportunistic infections, and any potential translation of pathway modulation in STAT1-GOF should therefore be approached cautiously. Future studies incorporating uninfected ruxolitinib-treated controls, together with fungal burden, histopathology, survival, and mechanistic readouts, will be required to determine whether pathway tuning can selectively improve dysregulated immunity without compromising antifungal host defense.

Our findings suggest several practical translational readouts for STAT1-GOF–associated fungal susceptibility. First, combining host-genetic diagnosis with functional immune assays (e.g., PBMC CFU-based outgrowth) may help stratify pathogen-specific vulnerability beyond cytokine measurements alone. Second, infection-time immune monitoring focused on interferon-biased states and IL-17–linked programs, together with innate-like lymphoid readouts (including NKT cells), may provide a mechanistically informed framework to interpret immune dysregulation during talaromycosis. Finally, although we used ruxolitinib as an experimental probe of pathway tunability rather than a therapeutic claim, the data motivates future studies to define whether carefully timed pathway modulation can reshape harmful immune remodeling without compromising fungal control, ideally alongside antifungal coverage and close microbiologic and immunologic monitoring.

This study has several limitations. The number of human donors was limited, and our mechanistic signaling assays relied on an overexpression system in HEK293T cells, which may not fully capture pathway regulation in primary immune compartments. In addition, intracellular vector copy number or transgene abundance was not directly quantified in the transient transfection experiments. Therefore, although equal plasmid input and Renilla normalization were used, we cannot fully exclude a contribution from variable plasmid uptake or expression level to the observed signaling outputs, and future studies incorporating quantitative assessment of transgene abundance or vector copy number will be required to address this directly. *In vivo* analyses focused on a single endpoint (16 dpi), and additional time points will be important to distinguish early immune programming from late-stage pathology. Moreover, while our data support associations between interferon hyperactivation, altered lung immune programs, and susceptibility, causal links among interferon bias, IL-17-associated axes, innate-like lymphoid function, and fungal control remain to be established. In addition, although ruxolitinib treatment modulated infection-associated MDSC expansion in our model, microbiologic or clinical endpoints under treatment conditions (e.g., fungal burden, survival, or histopathology) were not evaluated, and therefore the findings should be interpreted as evidence of immune remodeling rather than therapeutic efficacy. An additional limitation is that uninfected ruxolitinib-treated WT and STAT1-L351F groups were not included; therefore, the present study cannot determine whether the observed MDSC changes reflect correction of steady-state GOF-associated immune dysregulation or a more general effect of ruxolitinib on basal myeloid homeostasis. Moreover, although transcriptomic analyses suggested alterations in IL-17 associated pathways, functional validation of Th17 responses was not performed. Future studies incorporating intracellular IL-17A staining or T cell functional assays will be required to directly assess Th17 immunity in this model. Despite these limitations, our integrated human-to-animal framework provides a framework for understanding potential mechanisms for STAT1-L351F GOF-associated susceptibility to *T. marneffei* and highlights JAK–STAT pathway modulation as an experimentally tractable avenue to interrogate infection-associated immune remodeling (12, 27, 28, 30).

## 5 Conclusion

In summary, STAT1-L351F is associated with enhanced interferon signaling and is associated with increased susceptibility to *T. marneffei* infection in female mice, accompanied by lung immune remodeling and a type I interferon-biased but dysregulated antifungal transcriptional program. Proof-of-concept JAK inhibition in infected mice was associated with altered MDSC-related readouts, supporting the use of pathway perturbation as an experimental tool to interrogate infection-associated immune remodeling in STAT1-GOF-associated fungal susceptibility, while not establishing correction of steady-state immune abnormalities.

## Data Availability

The datasets analyzed for this study can be found in GEO, accession number: GSE328258.

## Supplementary material

Supplementary Table 1 Antibodies and staining conditions used for flow cytometry.Volumes indicate antibody amounts added per test as specified in the flow cytometry report (per tube containing ~1×10^6 cells in 100 µL). Blank cells indicate not used in that panel.

Supplementary Figure 1 Gating strategy for splenic MDSC subsets corresponding to **Figures 4B, C**. Representative spleen samples collected at 16 dpi are shown. Panels **(A–C)** depict the gating sequence for sample G48 (Stat1-L351F + *T. marneffei*), and panels **(D–F)** for sample G87 (Stat1-L351F + *T. marneffei* + ruxolitinib). Live cells were first gated for CD45^+^ leukocytes **(A, D)**, followed by gating of CD11b^+^ myeloid cells within CD45^+^ cells **(B, E)**. MDSC subsets were then defined within the CD11b^+^ gate based on Ly6G and Ly6C expression **(C, F)**, including granulocytic MDSCs (G-MDSCs; Ly6G^+^Ly6C^low) and monocytic MDSCs (M-MDSCs; Ly6G^-^Ly6C^high). Percentages indicate the frequency within the indicated parent gates. The same gating thresholds were applied across all samples after initial determination using FMO controls.

## Glossary

CFU: colony-forming units
dpi: days post-infection
GAS: gamma-activated sequence
GOF: gain-of-function
IFN: interferon
IFNAR: interferon-alpha/beta receptor
ISRE: interferon-stimulated response element
MDSC: myeloid-derived suppressor cell
NKT: natural killer T
PBMC: peripheral blood mononuclear cells
RUX: ruxolitinib
WT: wild type
