# Supplementary material for: STAT1-L351F is associated with enhanced interferon signaling and susceptibility to Talaromyces marneffei infection
Source: Front Immunol. 2026 Apr 20;17:1813775. doi: 10.3389/fimmu.2026.1813775 (PMC13137508; doi:10.3389/fimmu.2026.1813775)
Supplement: Supplementary Table 1 — Antibodies and staining conditions used for flow cytometry.Volumes indicate antibody amounts added per test as specified in the flow cytometry report (per tube containing ~1×10^6 cells in 100 µL). Blank cells indicate not used in that panel. [file Table1.docx]

Table S1. Antibodies and staining conditions used for flow cytometry

Volumes indicate antibody amounts added per test as specified in the flow cytometry report (per tube containing ~1×10^6 cells in 100 µL). Blank cells indicate not used in that panel.

| Marker | Fluorochrome | Staining | Supplier | Catalog no. | TNK/Mac/MDSC panel (µL) | Treg/Tfh/Tfr panel (µL) | Th17 panel (µL) |
| --- | --- | --- | --- | --- | --- | --- | --- |
| Viability dye | Zombie UV | Viability | BioLegend | 423107 | 0.1 | 0.1 | 0.1 |
| CD11b | Alexa Fluor 700 | Surface | BioLegend | 101222 | 0.5 |  |  |
| CD3 | APC/Cy7 | Surface | BioLegend | 100222 | 1.25 | 1.25 | 0.5 |
| CD45 | FITC | Surface | BioLegend | 157214 | 0.5 | 0.5 | 0.5 |
| CD49b (pan-NK) | APC | Surface | BioLegend | 108909 | 1.25 |  |  |
| F4/80 | BV421 | Surface | BioLegend | 123137 | 1.25 |  |  |
| Ly-6C | PE | Surface | BioLegend | 128007 | 1.25 |  |  |
| Ly-6G | PE/Cy5 | Surface | BioLegend | 127671 | 1.25 |  |  |
