# Supplementary figures and images for: STAT1-L351F is associated with enhanced interferon signaling and susceptibility to Talaromyces marneffei infection
Source: Front Immunol. 2026 Apr 20;17:1813775. doi: 10.3389/fimmu.2026.1813775 (PMC13137508; doi:10.3389/fimmu.2026.1813775)

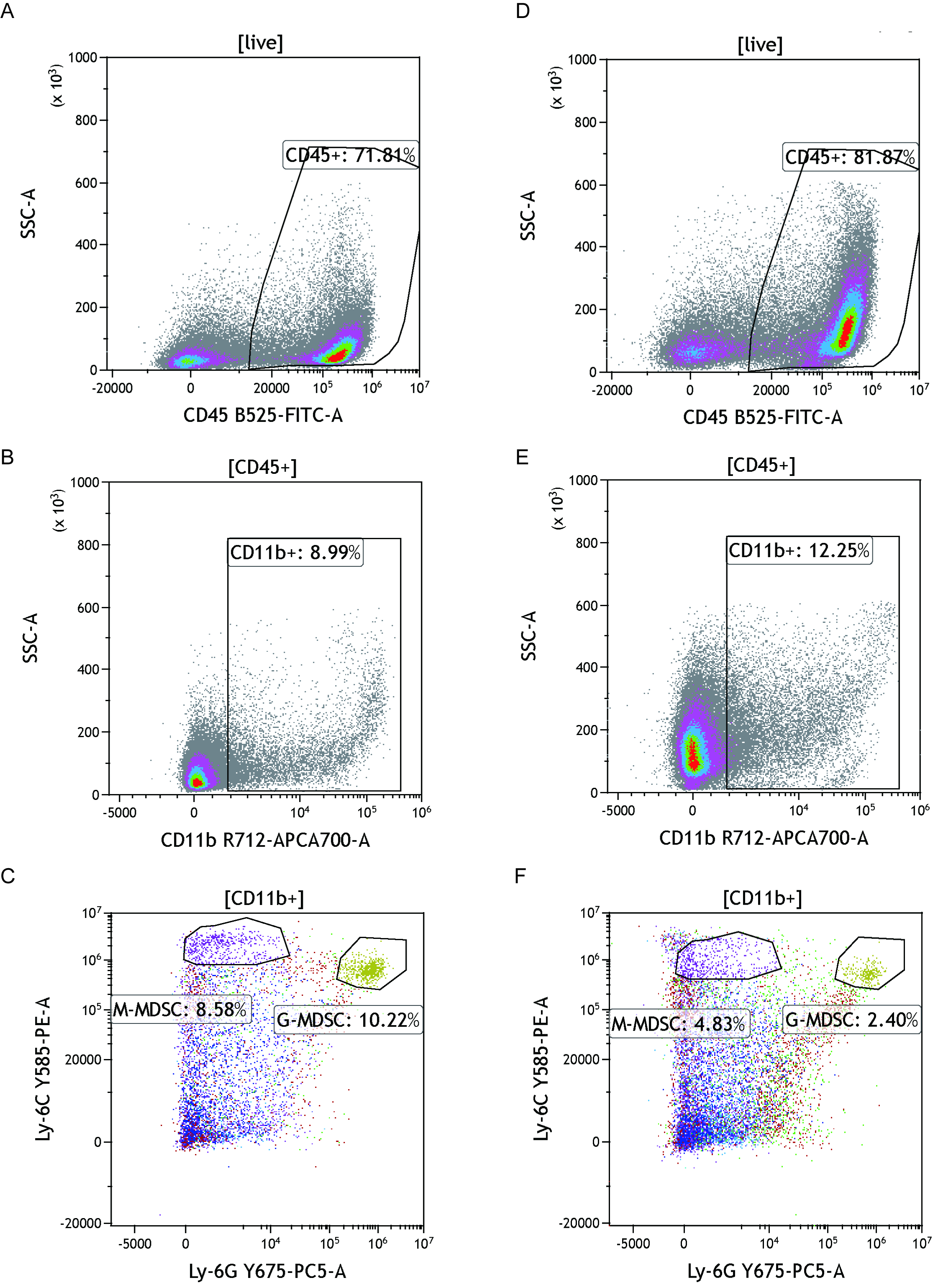

Supplement: Supplementary Figure 1 — Gating strategy for splenic MDSC subsets corresponding to Figures 4B, C. Representative spleen samples collected at 16 dpi are shown. Panels (A–C) depict the gating sequence for sample G48 (Stat1-L351F + T. marneffei), and panels (D–F) for sample G87 (Stat1-L351F + T. marneffei + ruxolitinib). Live cells were first gated for CD45+ leukocytes (A, D), followed by gating of CD11b+ myeloid cells within CD45+ cells (B, E). MDSC subsets were then defined within the CD11b+ gate based on Ly6G and Ly6C expression (C, F), including granulocytic MDSCs (G-MDSCs; Ly6G+Ly6C^low) and monocytic MDSCs (M-MDSCs; Ly6G-Ly6C^high). Percentages indicate the frequency within the indicated parent gates. The same gating thresholds were applied across all samples after initial determination using FMO controls. [file Image1.tif]

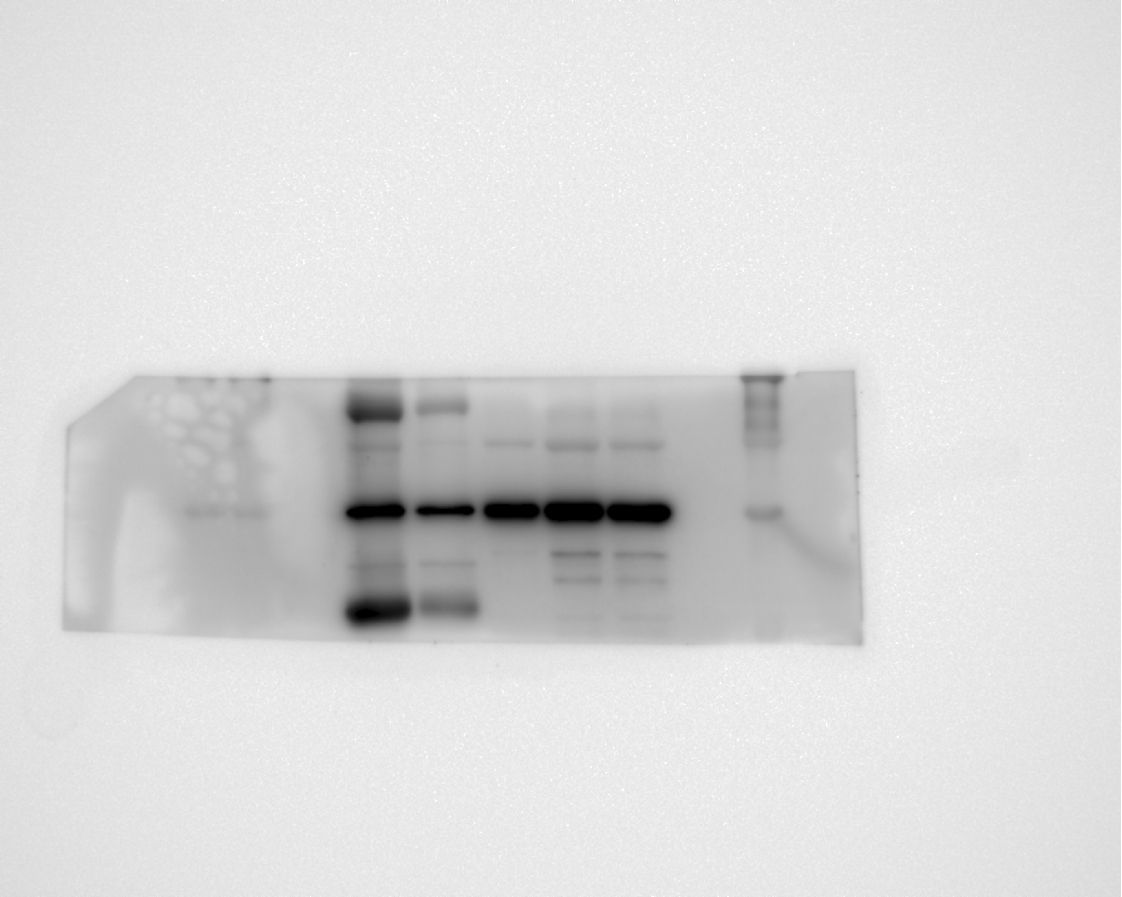

Supplement: Supplementary file 3 [file DataSheet1.zip › full uncropped Gels and Blots image(s)/Figure1-N1/LDL 2021-05-26 GAPDH-12s(Chemiluminescence).jpg]

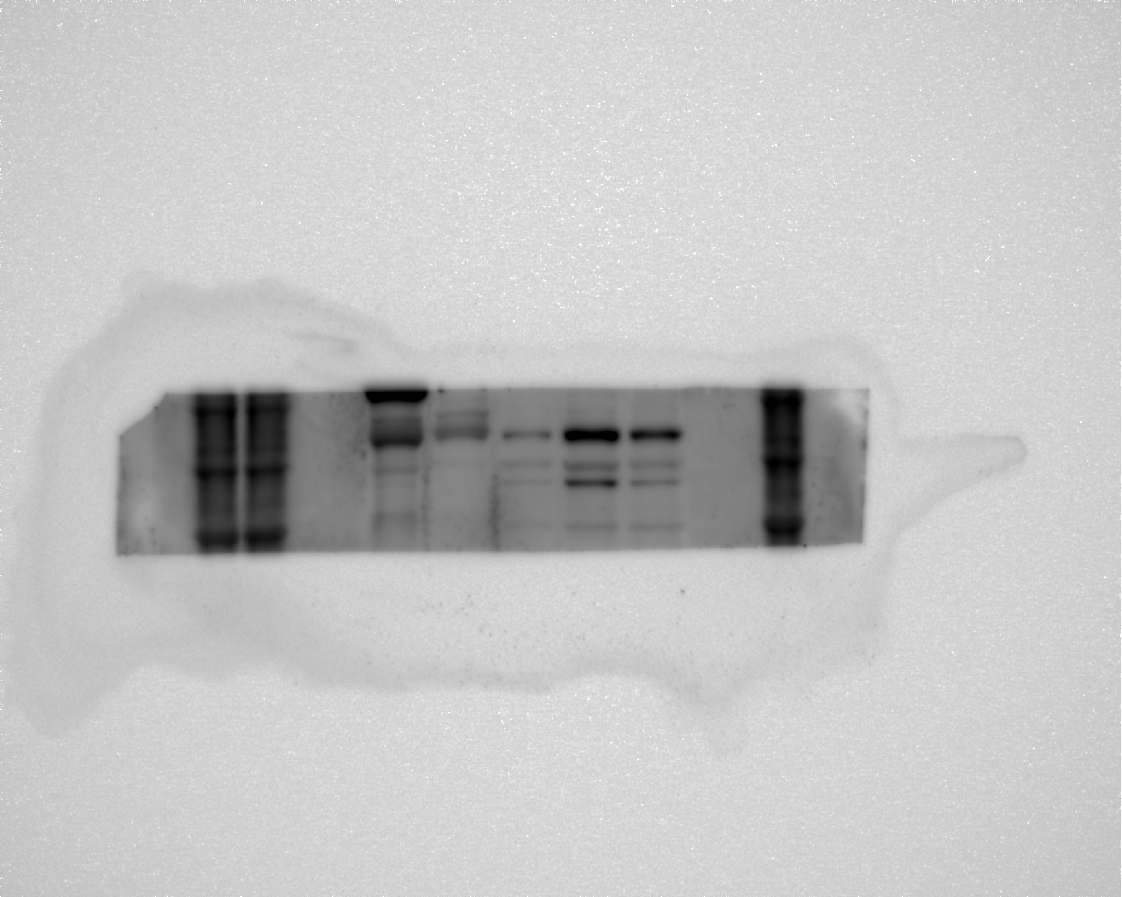

Supplement: Supplementary file 3 [file DataSheet1.zip › full uncropped Gels and Blots image(s)/Figure1-N1/LDL 2021-05-26 IFNAR2-25s(Chemiluminescence).jpg]

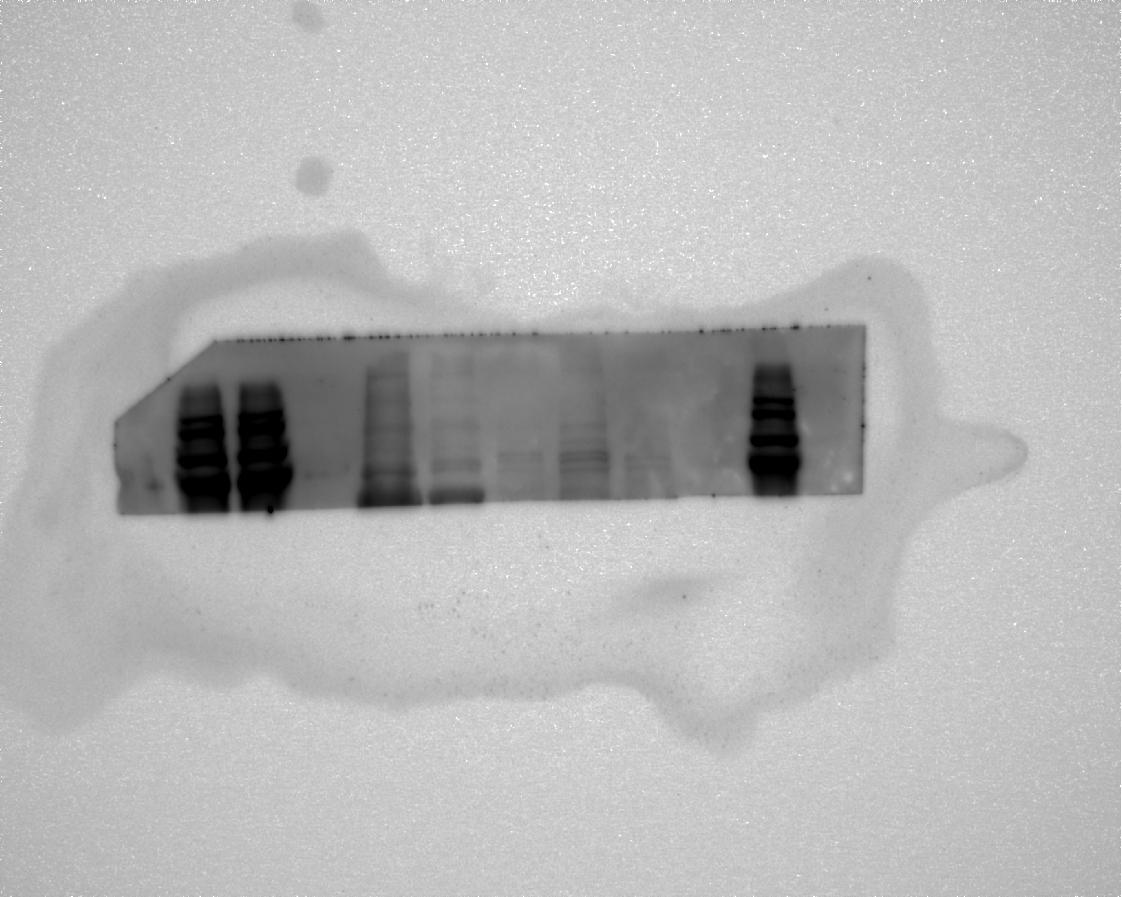

Supplement: Supplementary file 3 [file DataSheet1.zip › full uncropped Gels and Blots image(s)/Figure1-N1/LDL 2021-05-26 stat1-25s(Chemiluminescence).jpg]

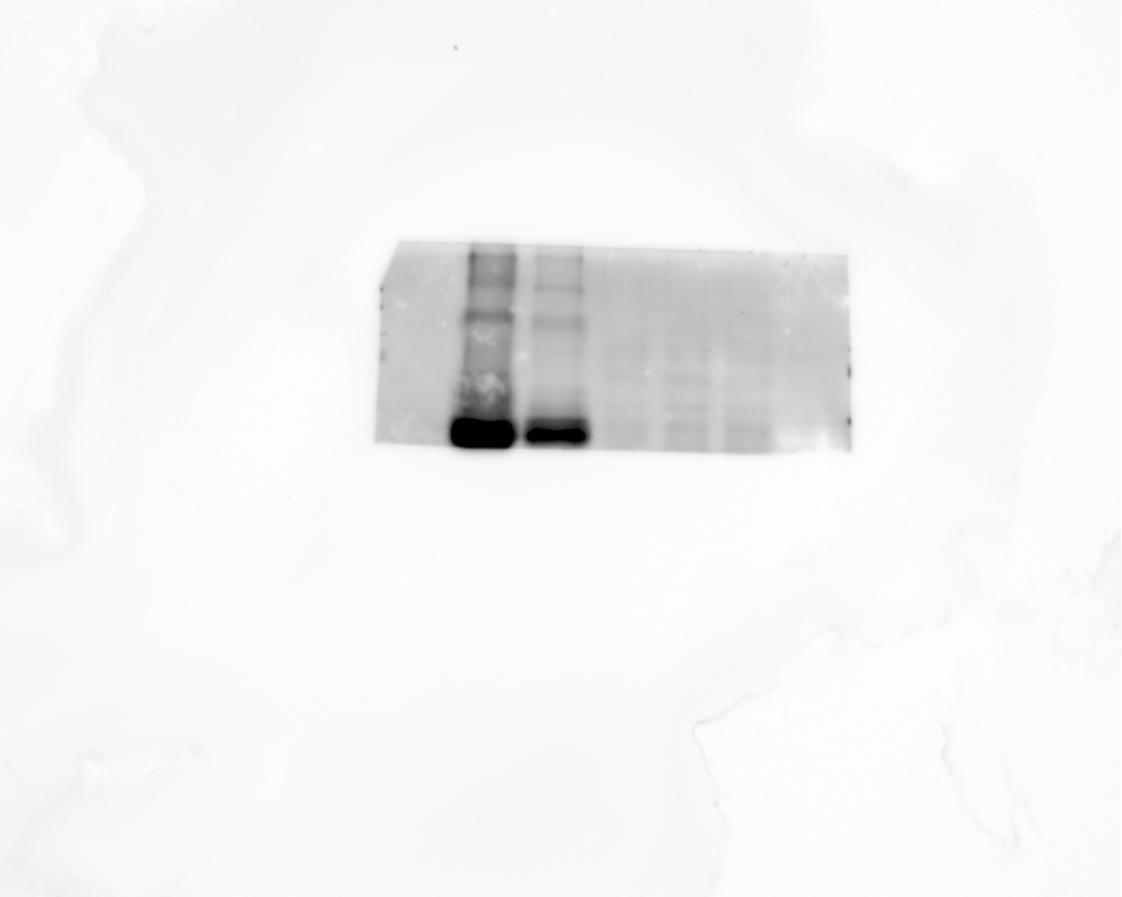

Supplement: Supplementary file 3 [file DataSheet1.zip › full uncropped Gels and Blots image(s)/Figure1-N1/LDL 2021-5-26 p-stat1-60s(Chemiluminescence).jpg]

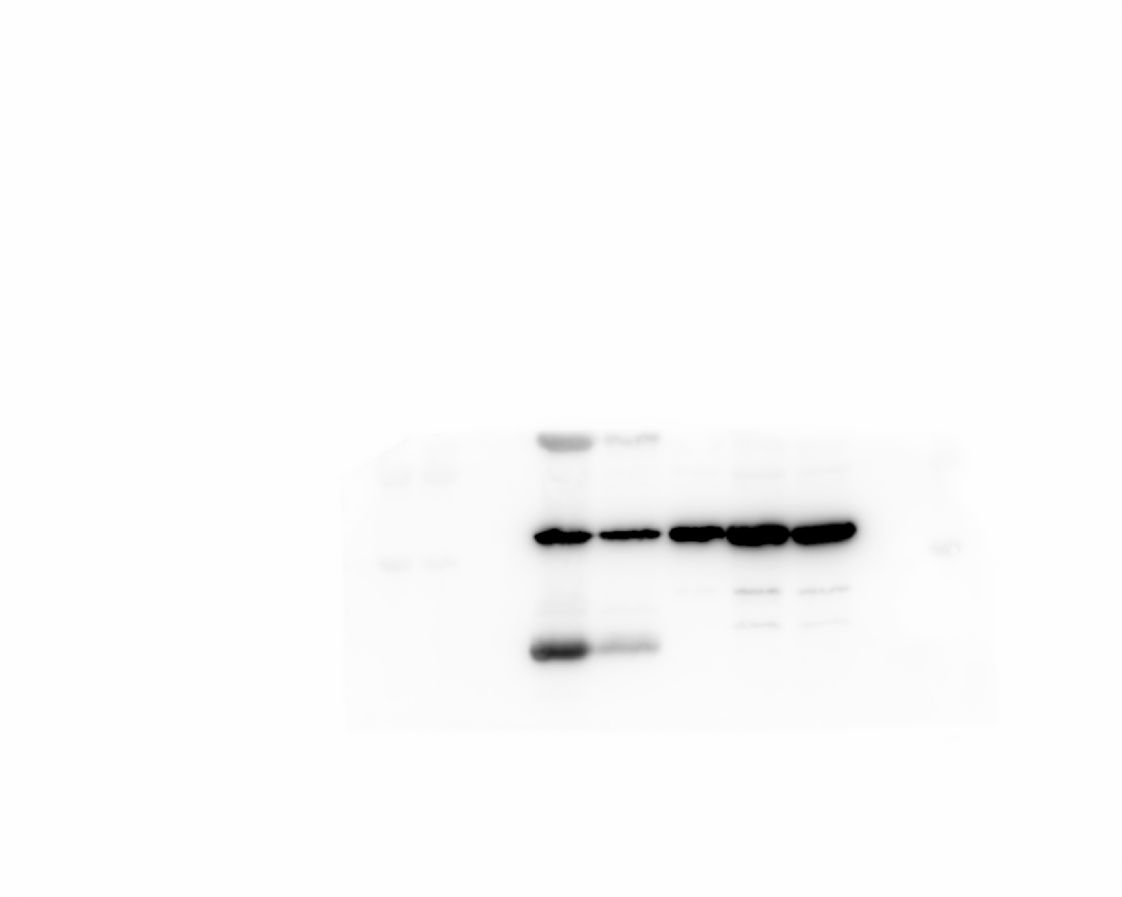

Supplement: Supplementary file 3 [file DataSheet1.zip › full uncropped Gels and Blots image(s)/Figure1-N2/LDL 2021-05-28 GAPDh-8s(Chemiluminescence).jpg]

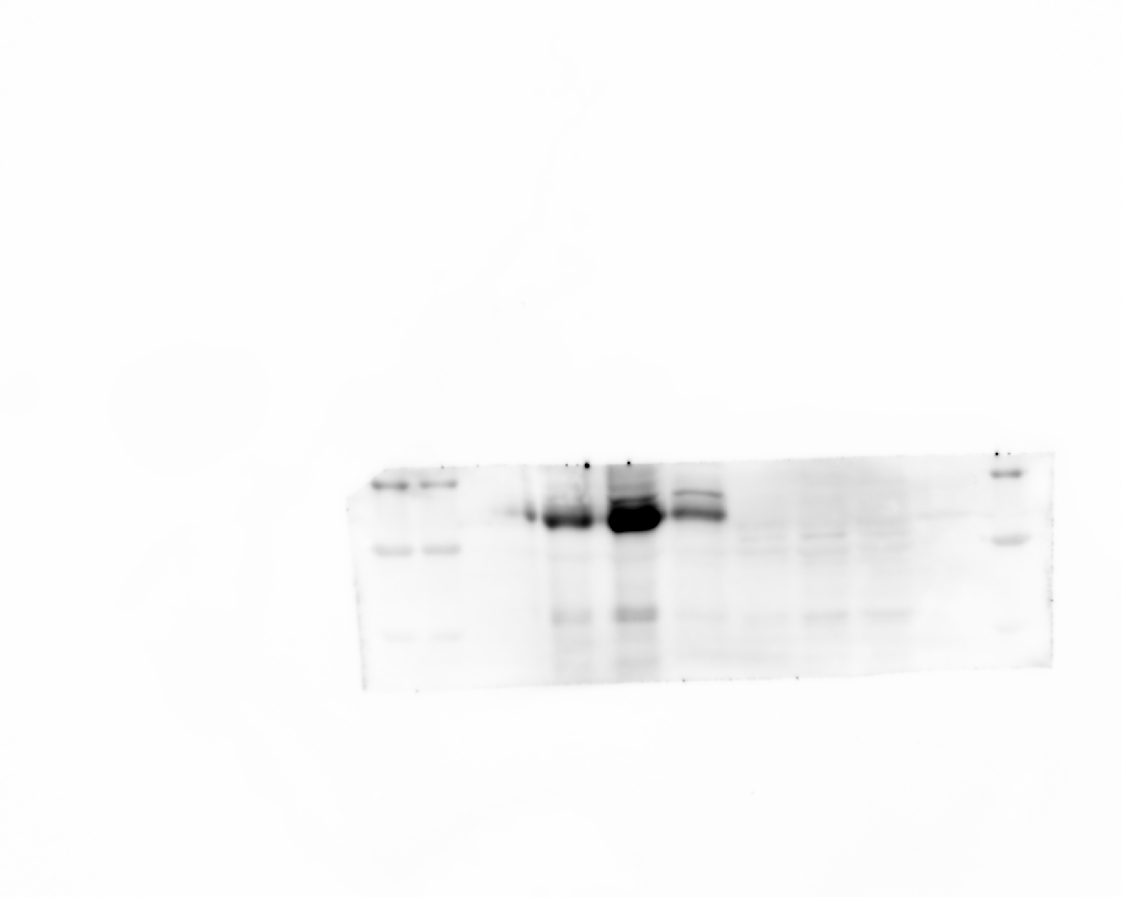

Supplement: Supplementary file 3 [file DataSheet1.zip › full uncropped Gels and Blots image(s)/Figure1-N2/LDL 2021-05-28 IFR9-30s(Chemiluminescence).jpg]

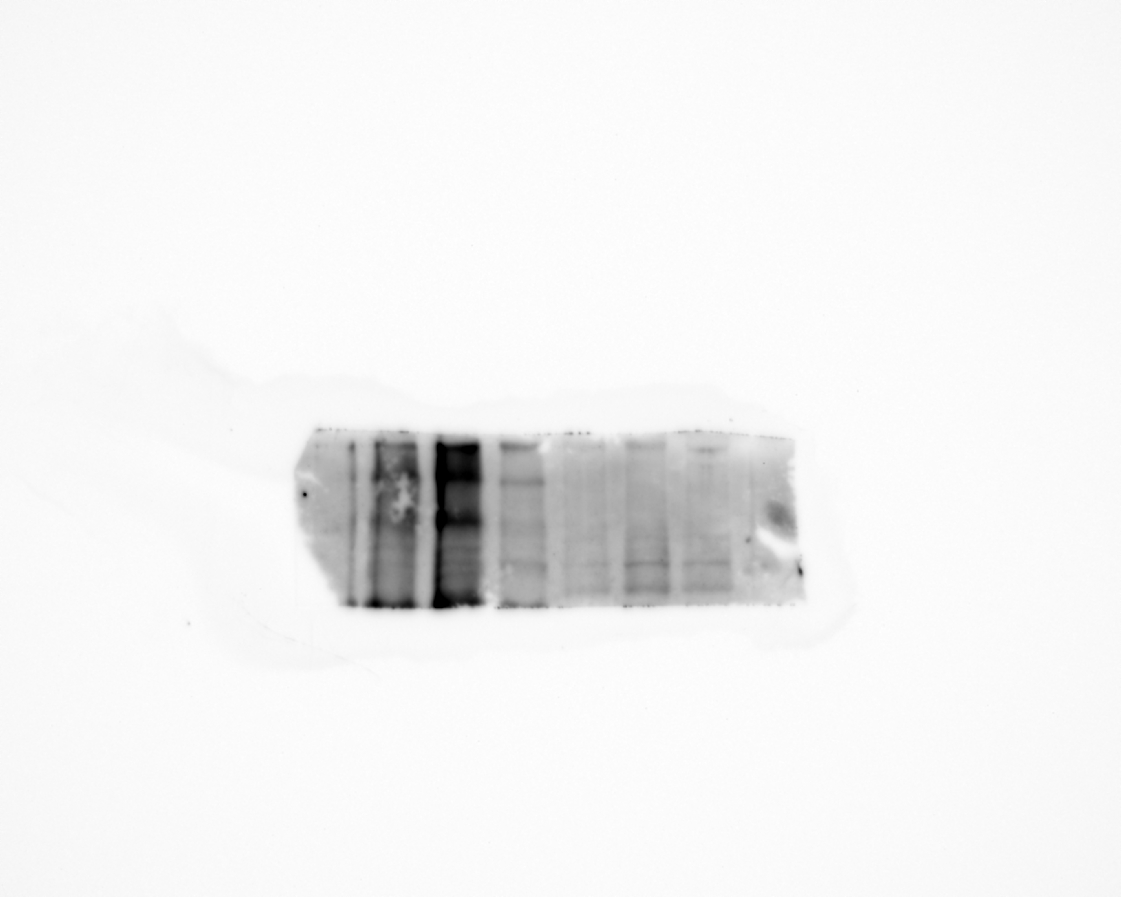

Supplement: Supplementary file 3 [file DataSheet1.zip › full uncropped Gels and Blots image(s)/Figure1-N2/LDL 2021-05-28 pstat2-120s(Chemiluminescence).jpg]

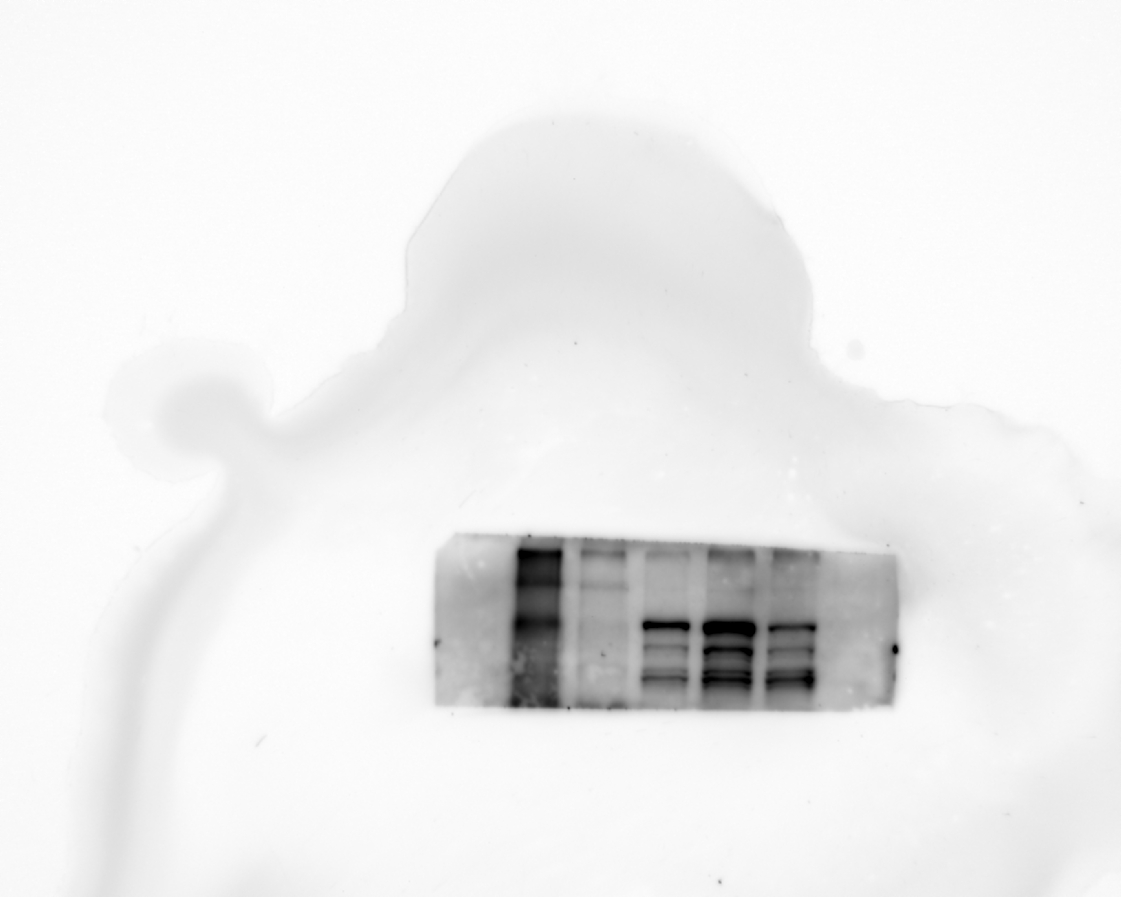

Supplement: Supplementary file 3 [file DataSheet1.zip › full uncropped Gels and Blots image(s)/Figure1-N2/LDL 2021-05-28stat2-120s(Chemiluminescence).jpg]

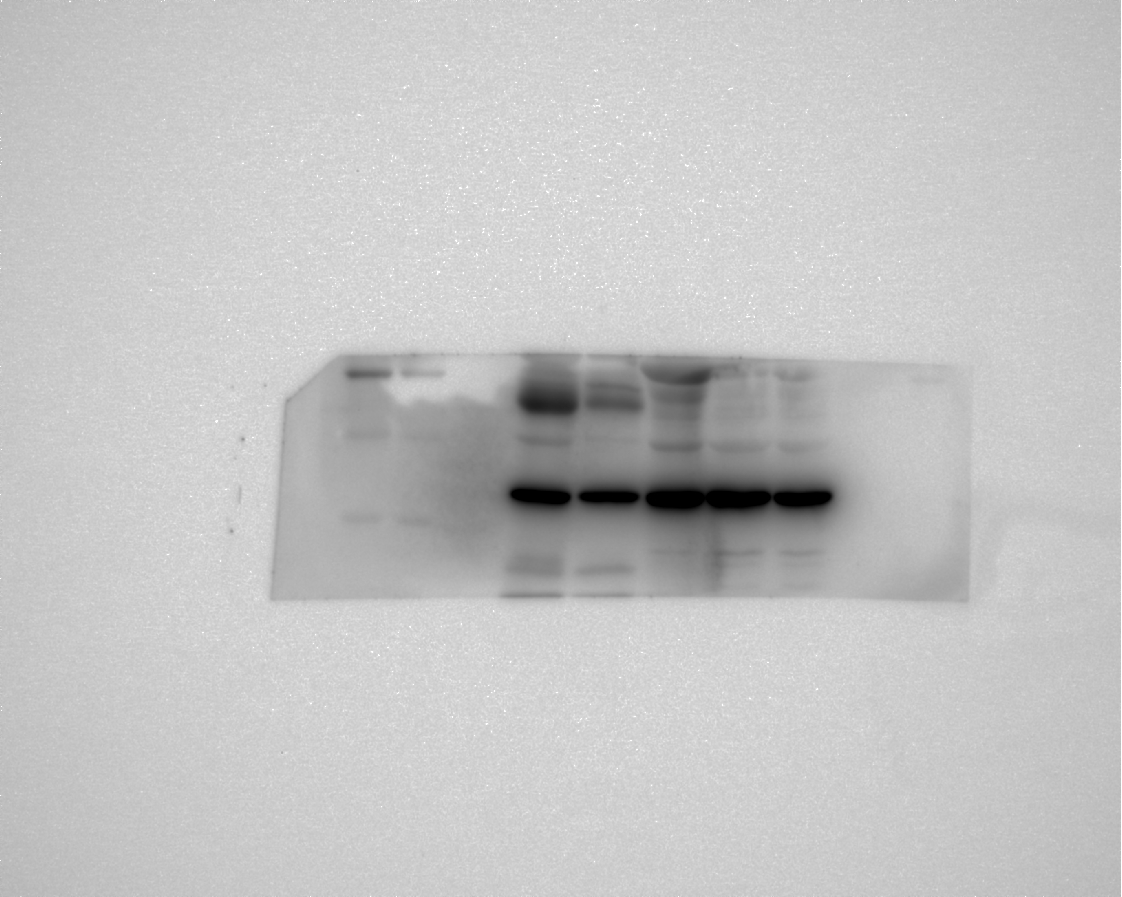

Supplement: Supplementary file 3 [file DataSheet1.zip › full uncropped Gels and Blots image(s)/Figure1-N3/LDL 2021-5-28 GAPDH-8s(Chemiluminescence).jpg]

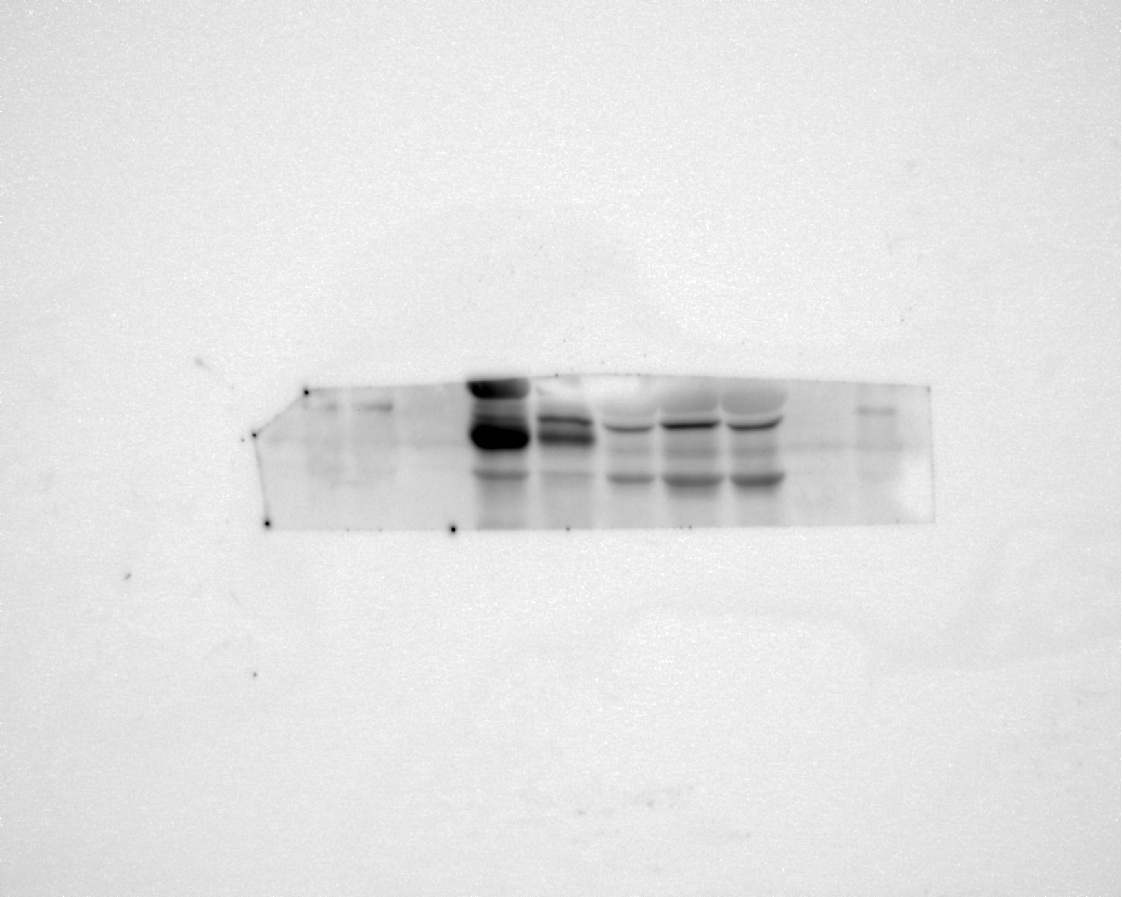

Supplement: Supplementary file 3 [file DataSheet1.zip › full uncropped Gels and Blots image(s)/Figure1-N3/LDL 2021-5-28 IFIT1-15s(Chemiluminescence).jpg]

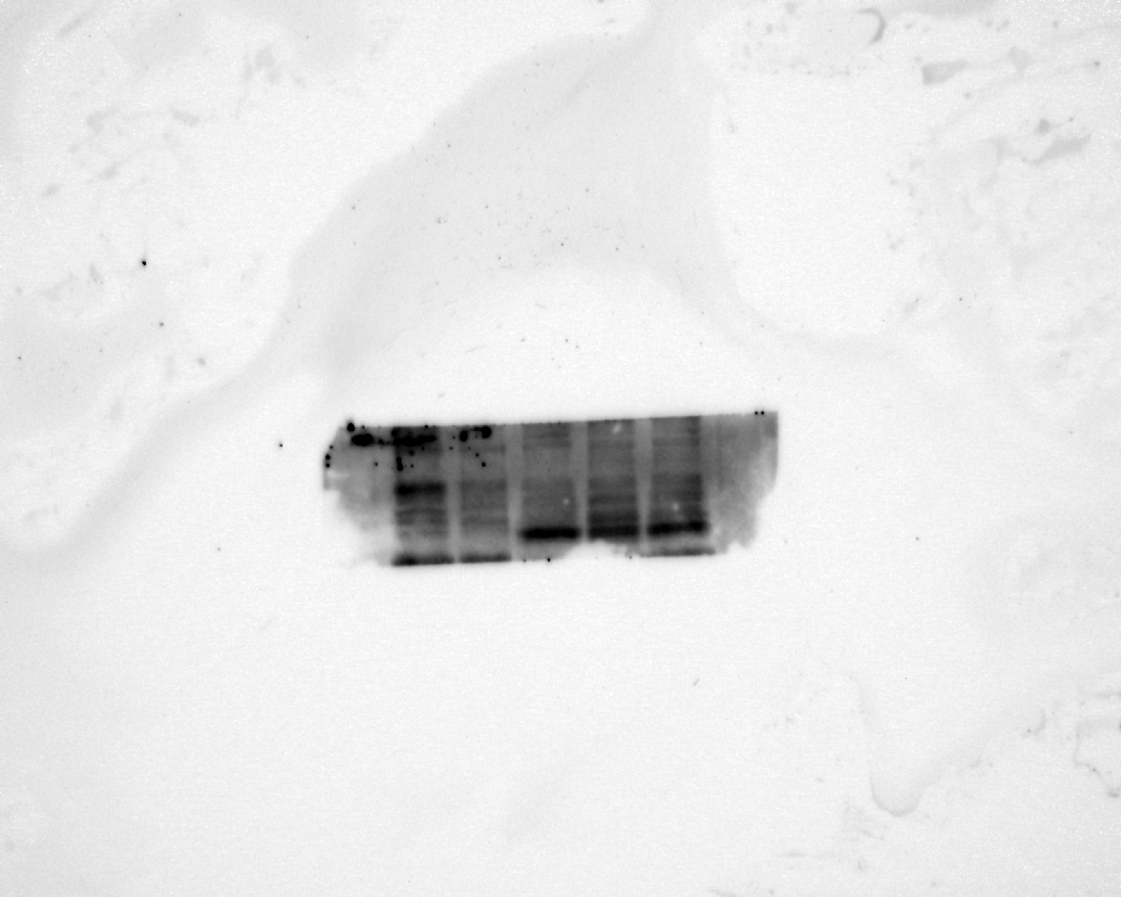

Supplement: Supplementary file 3 [file DataSheet1.zip › full uncropped Gels and Blots image(s)/Figure1-N3/LDL 2021-5-28 P-TYK2--60s(Chemiluminescence).jpg]

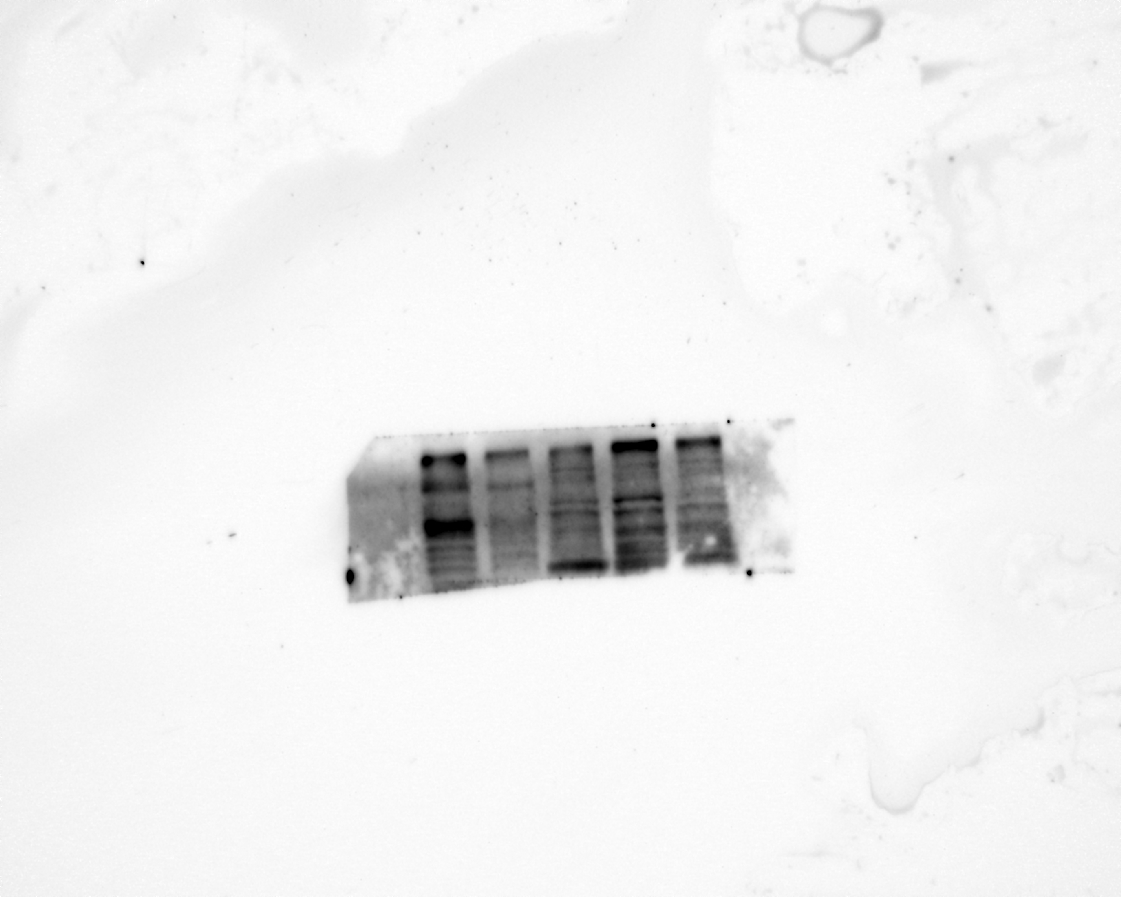

Supplement: Supplementary file 3 [file DataSheet1.zip › full uncropped Gels and Blots image(s)/Figure1-N3/LDL 2021-5-28 TYK2-120s(Chemiluminescence).jpg]

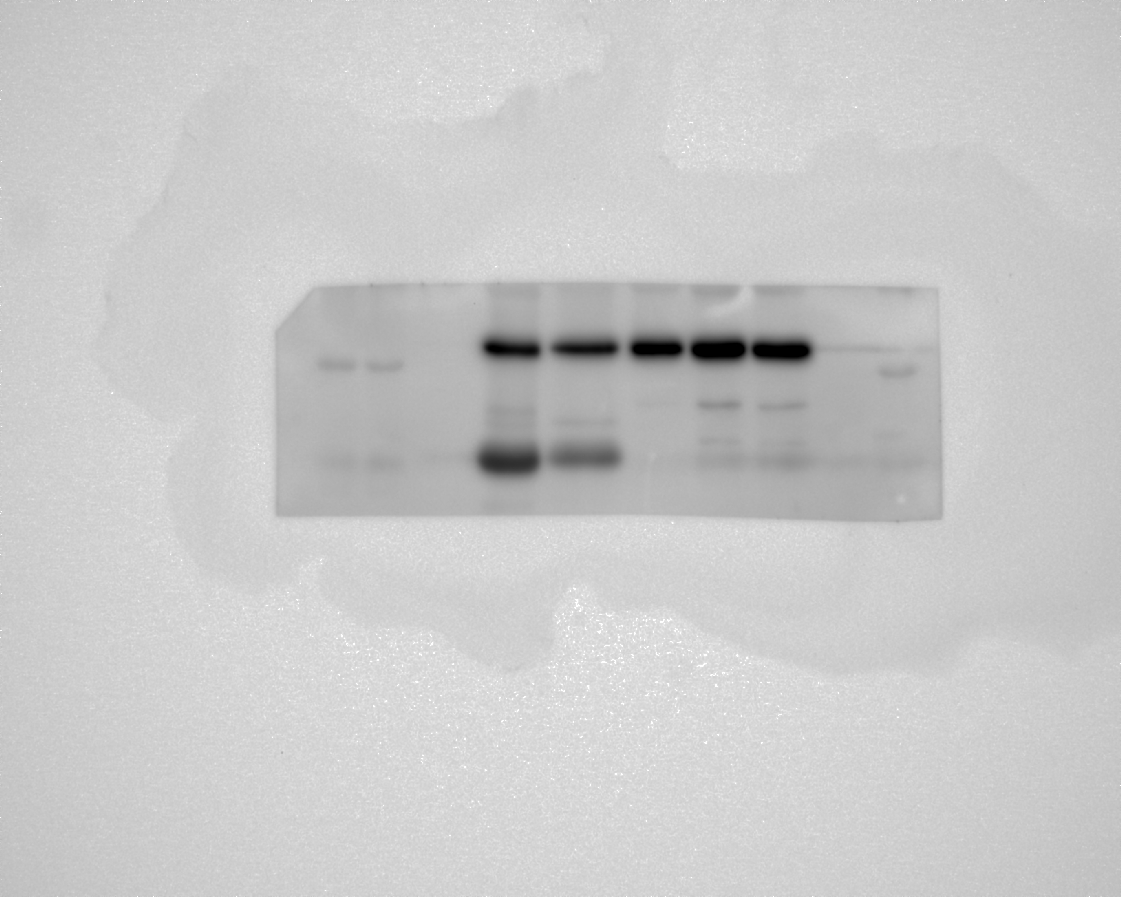

Supplement: Supplementary file 3 [file DataSheet1.zip › full uncropped Gels and Blots image(s)/Figure1-N4/LDL 2021-5-30 GAPDH-1-8s(Chemiluminescence).jpg]

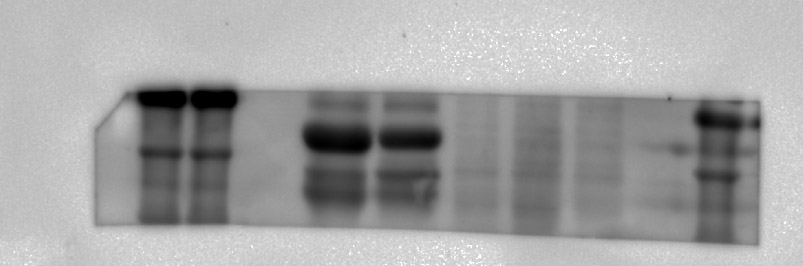

Supplement: Supplementary file 3 [file DataSheet1.zip › full uncropped Gels and Blots image(s)/Figure1-N4/LDL 2021-5-30 IFNAR1-15s(Chemiluminescence).jpg]

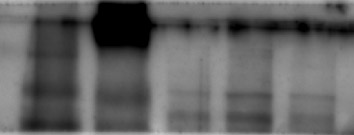

Supplement: Supplementary file 3 [file DataSheet1.zip › full uncropped Gels and Blots image(s)/Figure1-N4/LDL 2021-5-30 JAK1-60s(Chemiluminescence).jpg]

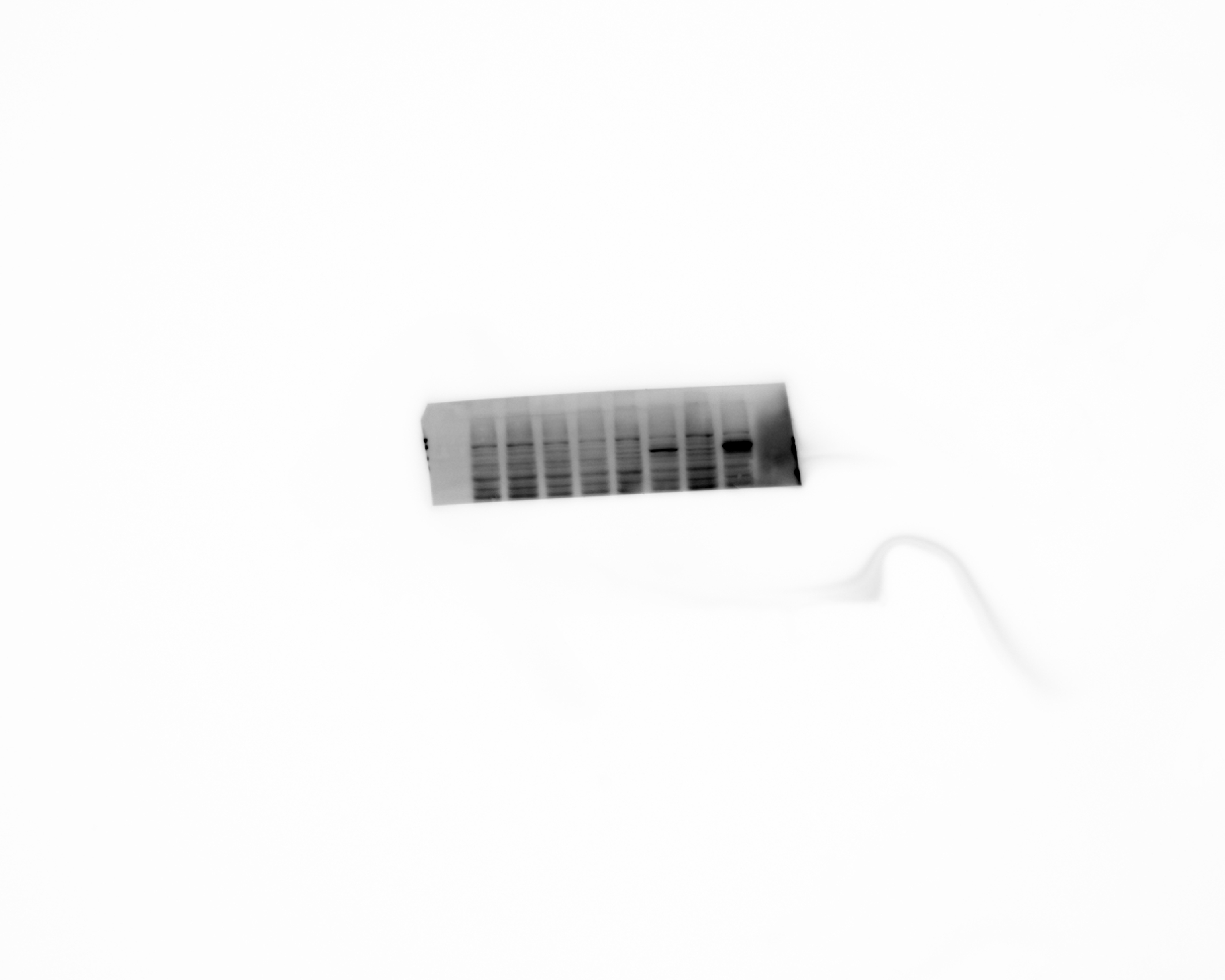

Supplement: Supplementary file 3 [file DataSheet1.zip › full uncropped Gels and Blots image(s)/Figure2-A/LDL 2022-01-28 stat1-35s(Chemiluminescence).jpg]

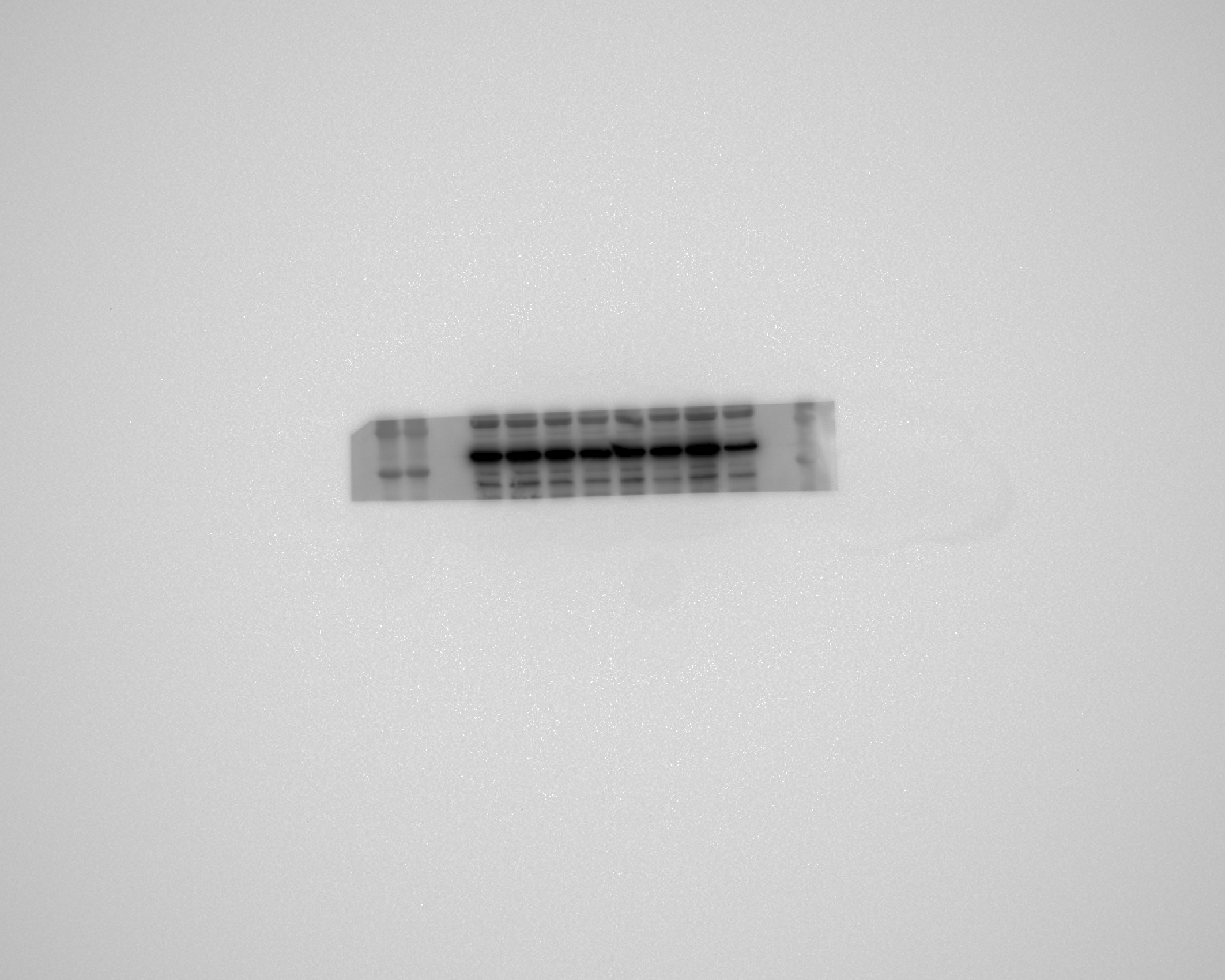

Supplement: Supplementary file 3 [file DataSheet1.zip › full uncropped Gels and Blots image(s)/Figure2-A/LDL 2022-1-26 GAPDH-5s(Chemiluminescence).jpg]

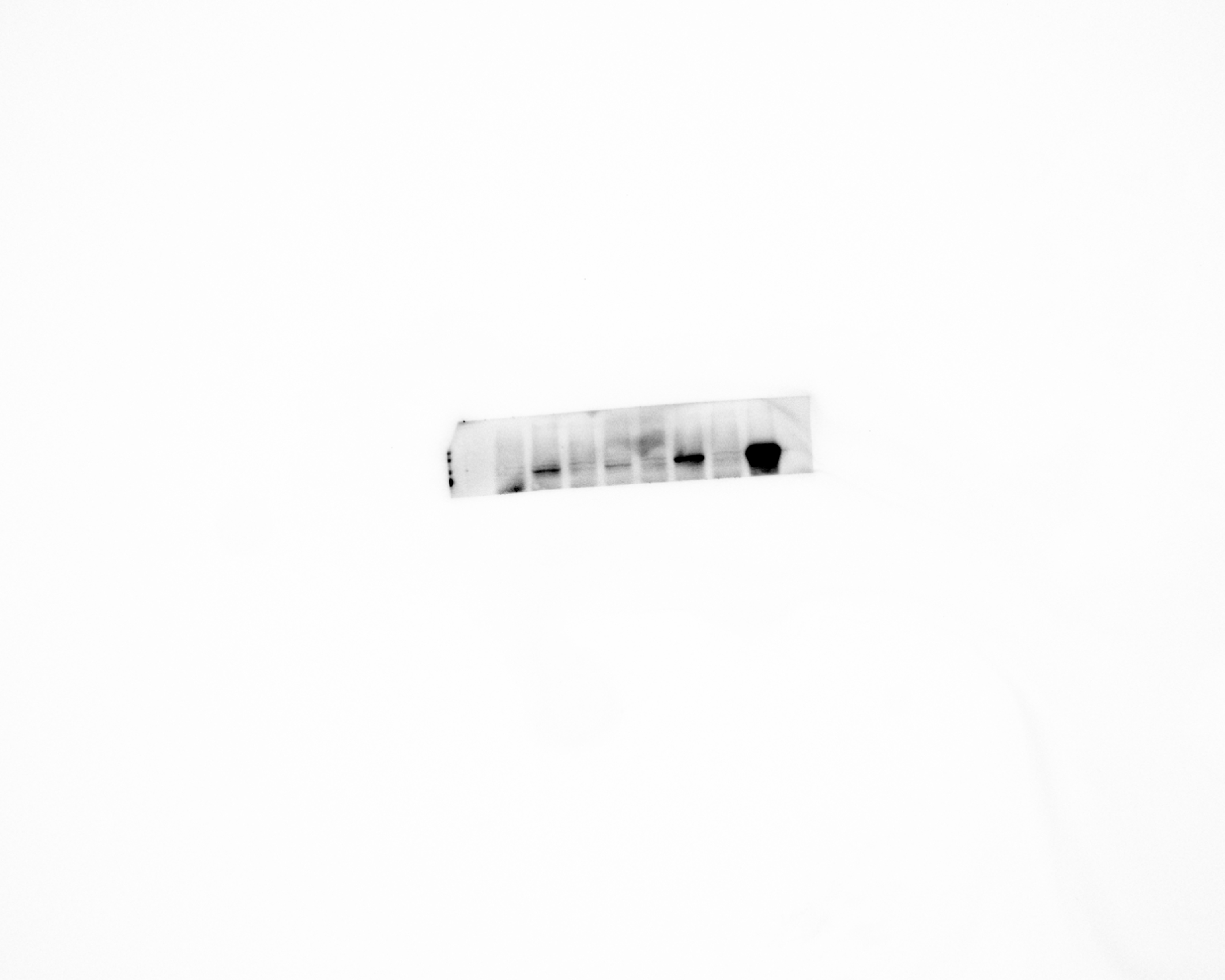

Supplement: Supplementary file 3 [file DataSheet1.zip › full uncropped Gels and Blots image(s)/Figure2-A/LDL 2022-1-26 p-stat1-15s(Chemiluminescence).jpg]
