# Supplementary material for: STAT1-L351F is associated with enhanced interferon signaling and susceptibility to Talaromyces marneffei infection
Source: Front Immunol. 2026 Apr 20;17:1813775. doi: 10.3389/fimmu.2026.1813775 (PMC13137508; doi:10.3389/fimmu.2026.1813775)
Supplement: Supplementary file 4 [file DataSheet2.pdf]

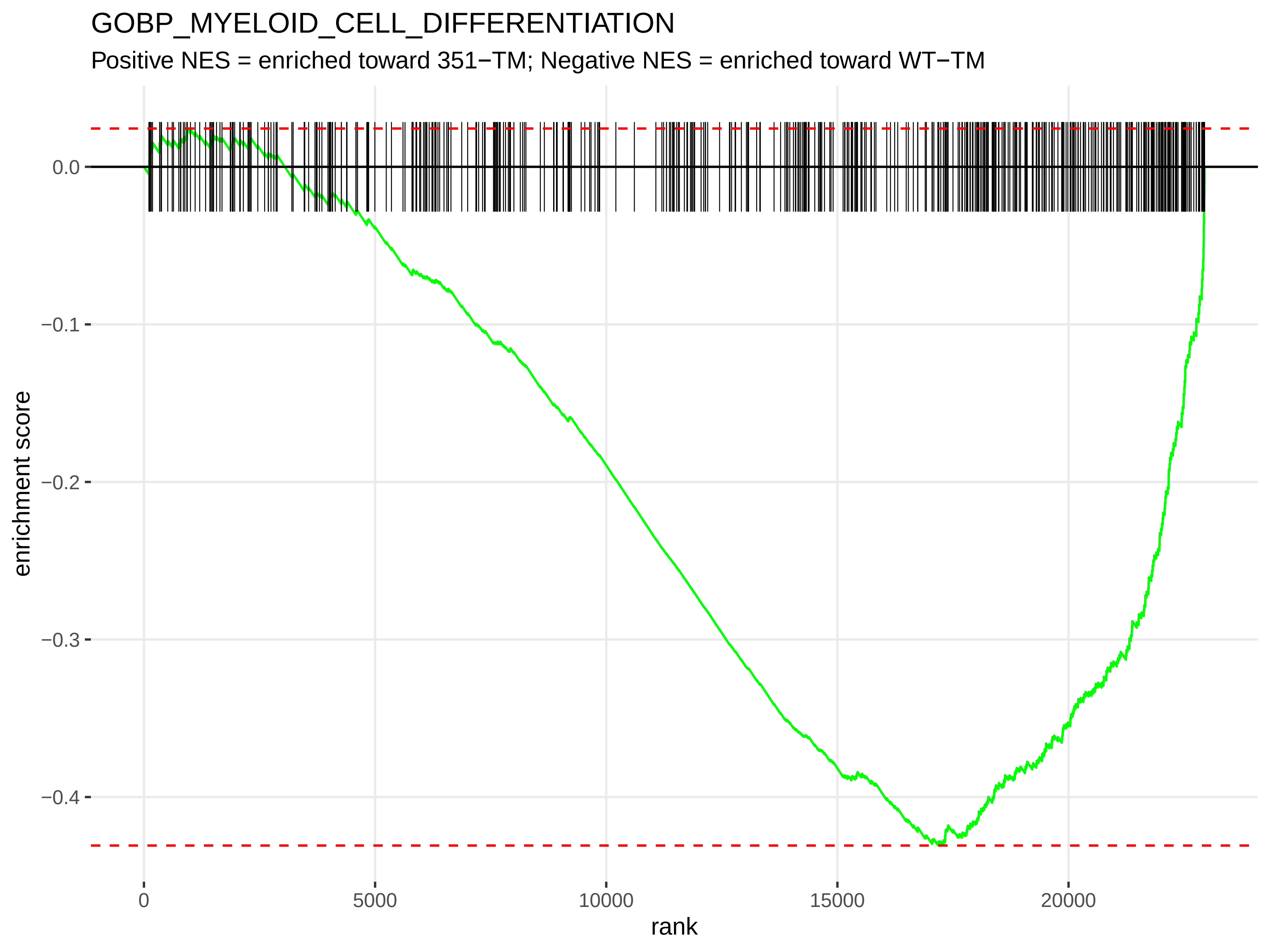
GOBP_MYELOID_CELL_DIFFERENTIATION
Positive NES = enriched toward 351−TM; Negative NES = enriched toward WT−TM
0.0
−0.1
−0.2
−0.3
−0.4
enrichment score
0
5000
10000
15000
20000
rank

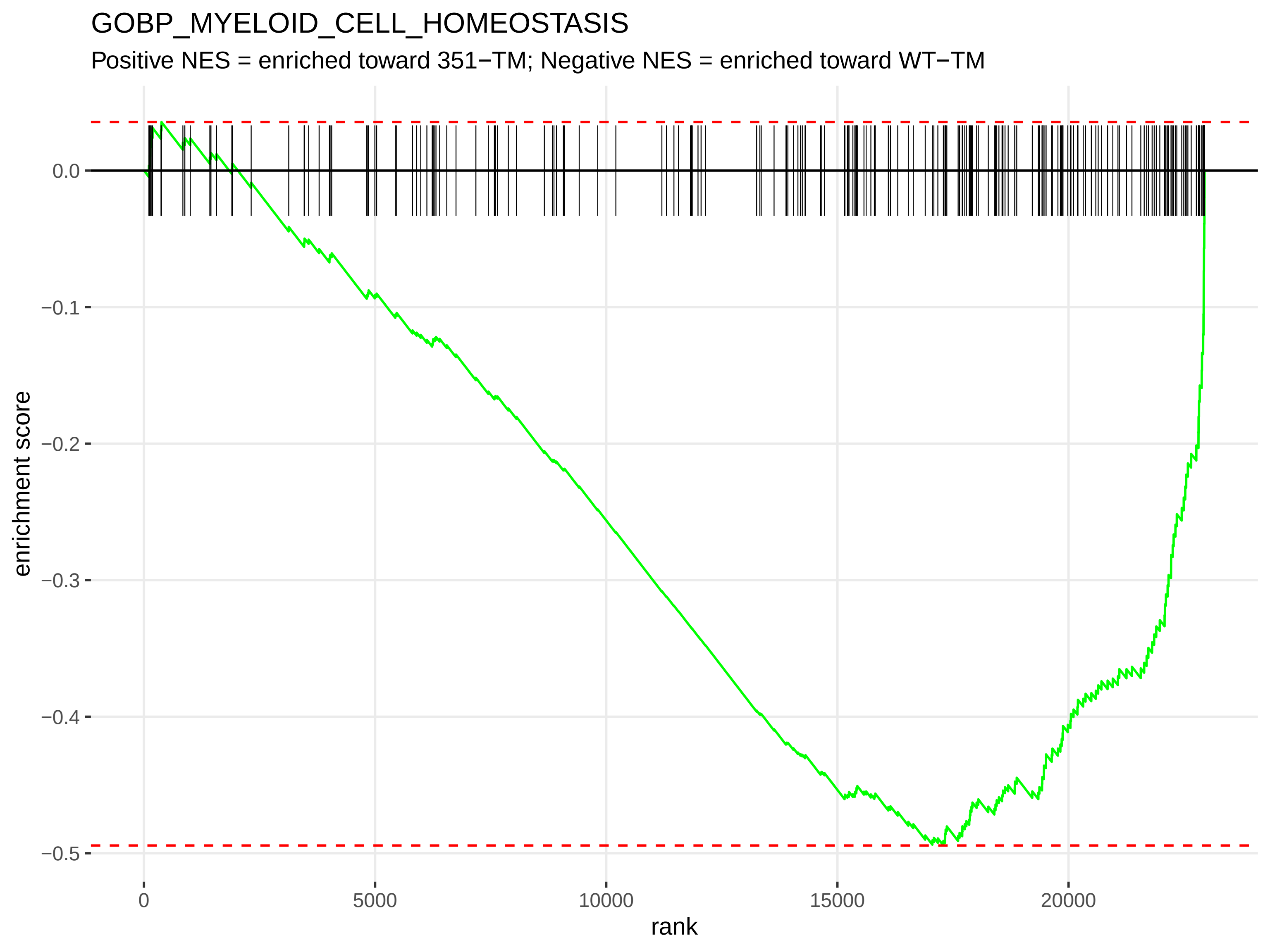
GOBP_MYELOID_CELL_HOMEOSTASIS
Positive NES = enriched toward 351−TM; Negative NES = enriched toward WT−TM
enrichment score
0.0
−0.1
−0.2
−0.3
−0.4
−0.5
0
5000
10000
15000
20000
rank

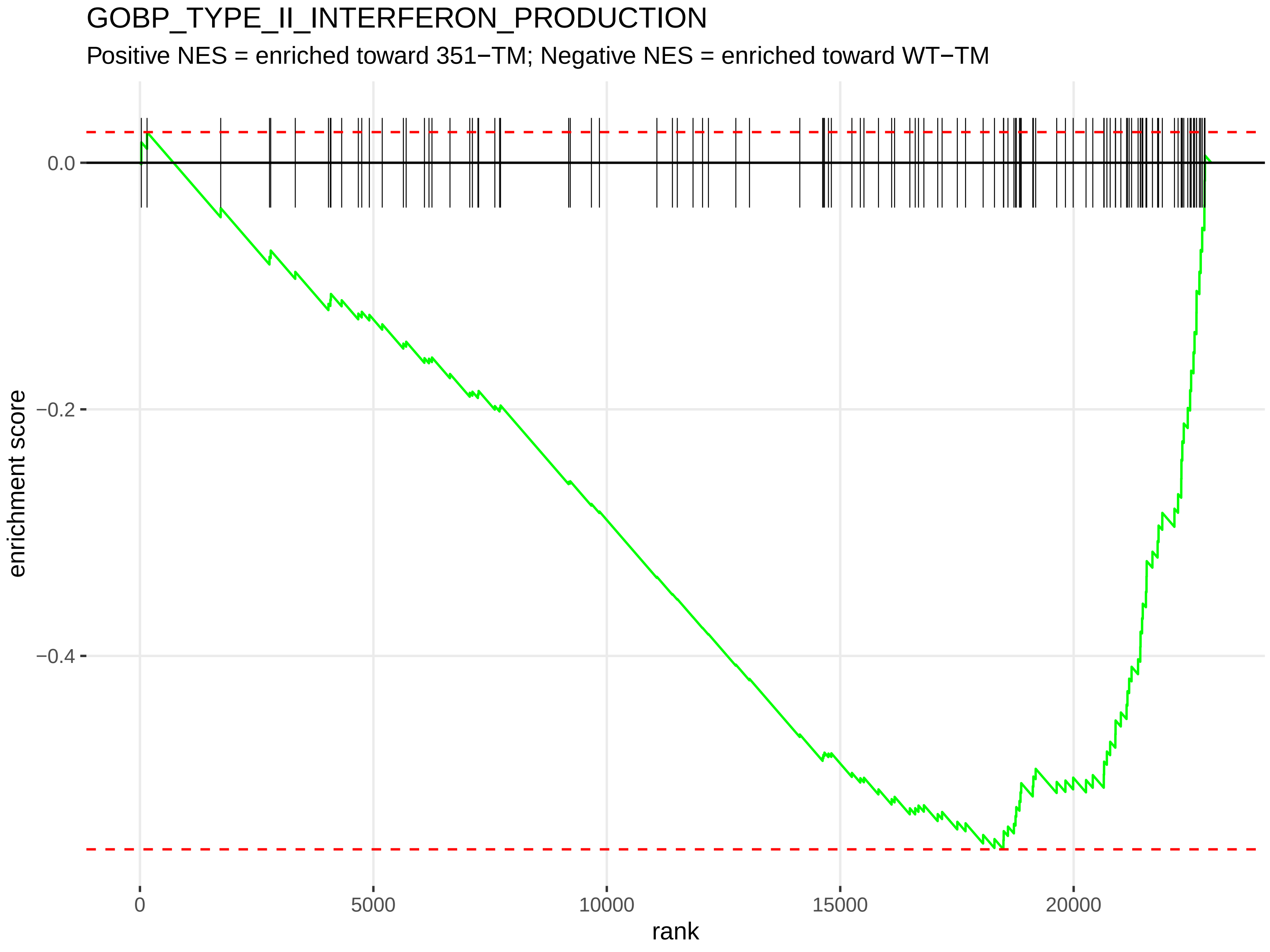
GOBP_TYPE_II_INTERFERON_PRODUCTION
Positive NES = enriched toward 351−TM; Negative NES = enriched toward WT−TM
enrichment score
0.0
−0.2
−0.4
0
5000
10000
15000
20000
rank

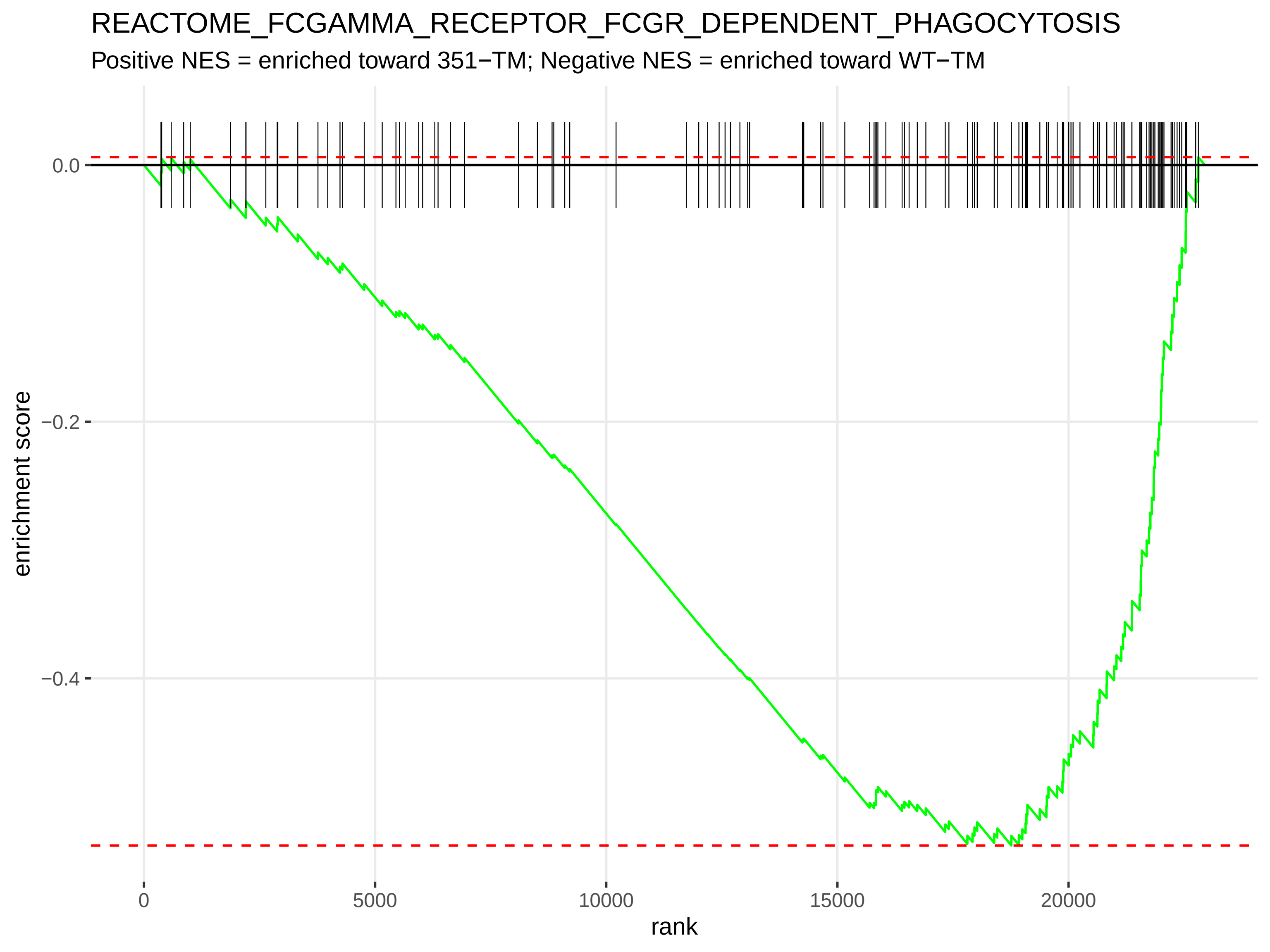

REACTOME_FCGAMMA_RECEPTOR_FCGR_DEPENDENT_PHAGOCYTOSIS
Positive NES = enriched toward 351−TM; Negative NES = enriched toward WT−TM
enrichment score
0.0
−0.2
−0.4
0
5000
10000
15000
20000
rank

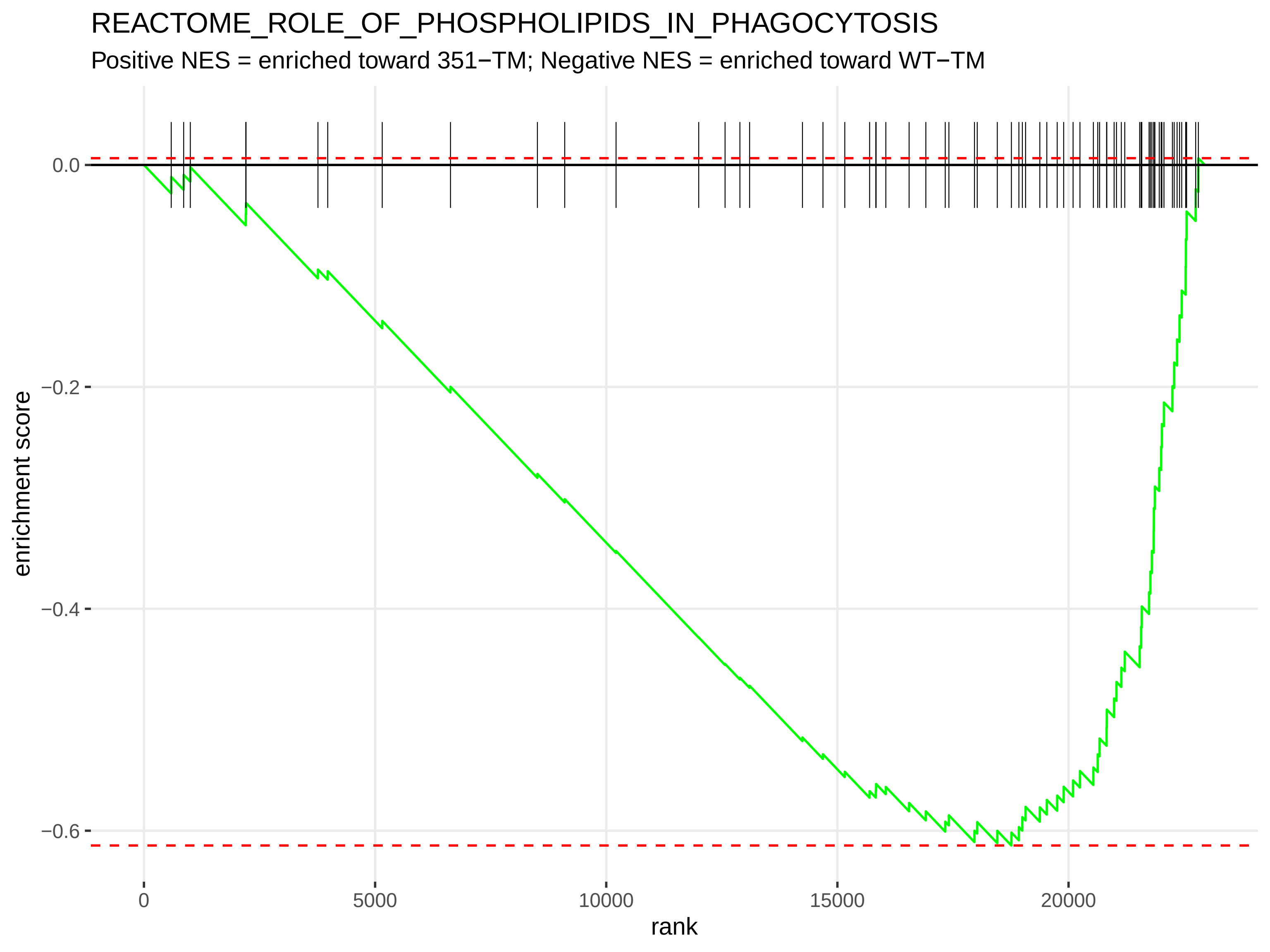
REACTOME_ROLE_OF_PHOSPHOLIPIDS_IN_PHAGOCYTOSIS
Positive NES = enriched toward 351−TM; Negative NES = enriched toward WT−TM
enrichment score
0.0
−0.2
−0.4
−0.6
0
5000
10000
15000
20000
rank

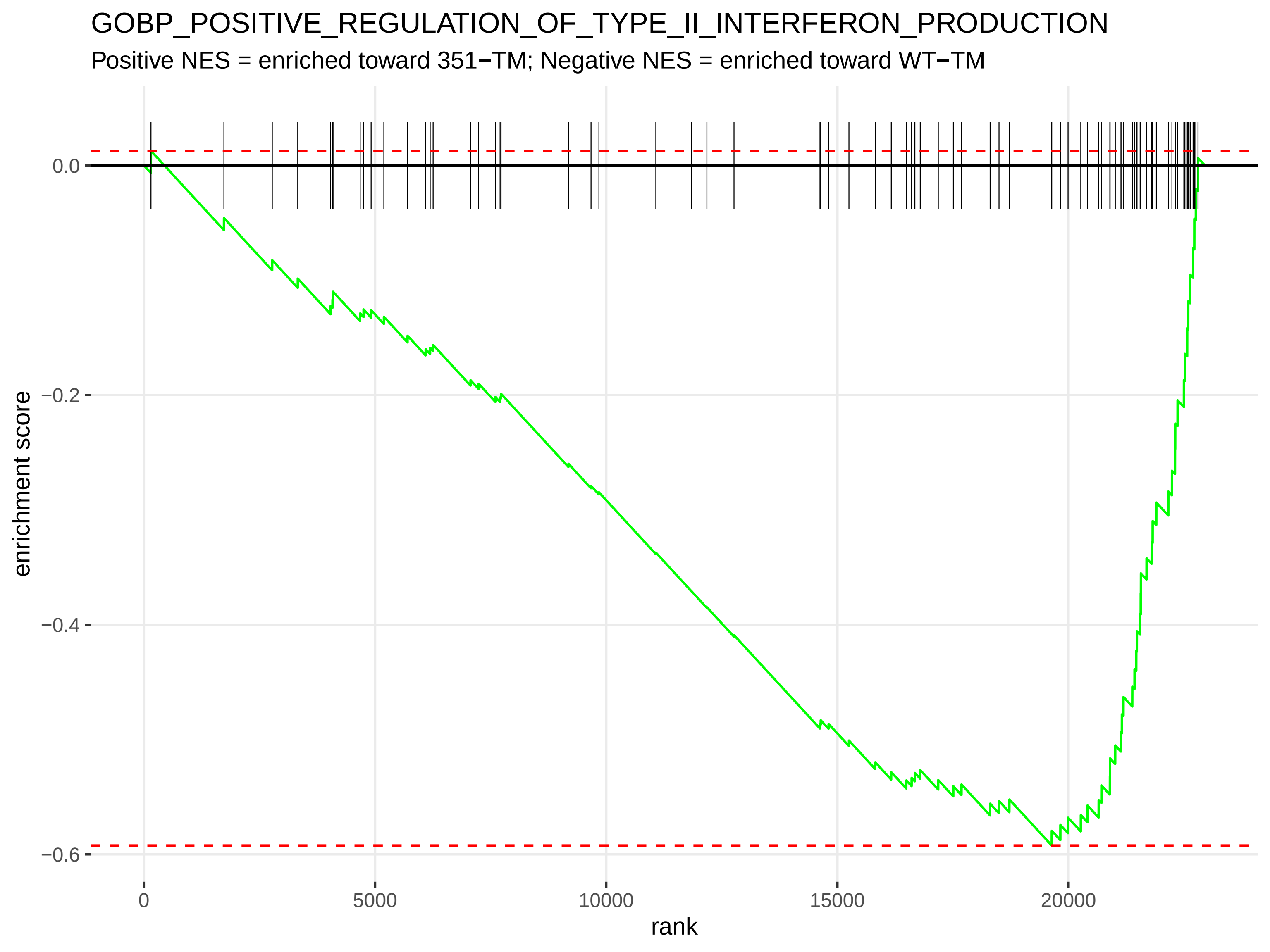
GOBP_POSITIVE_REGULATION_OF_TYPE_II_INTERFERON_PRODUCTION
Positive NES = enriched toward 351−TM; Negative NES = enriched toward WT−TM
enrichment score
0.0
−0.2
−0.4
−0.6
0
5000
10000
15000
20000
rank

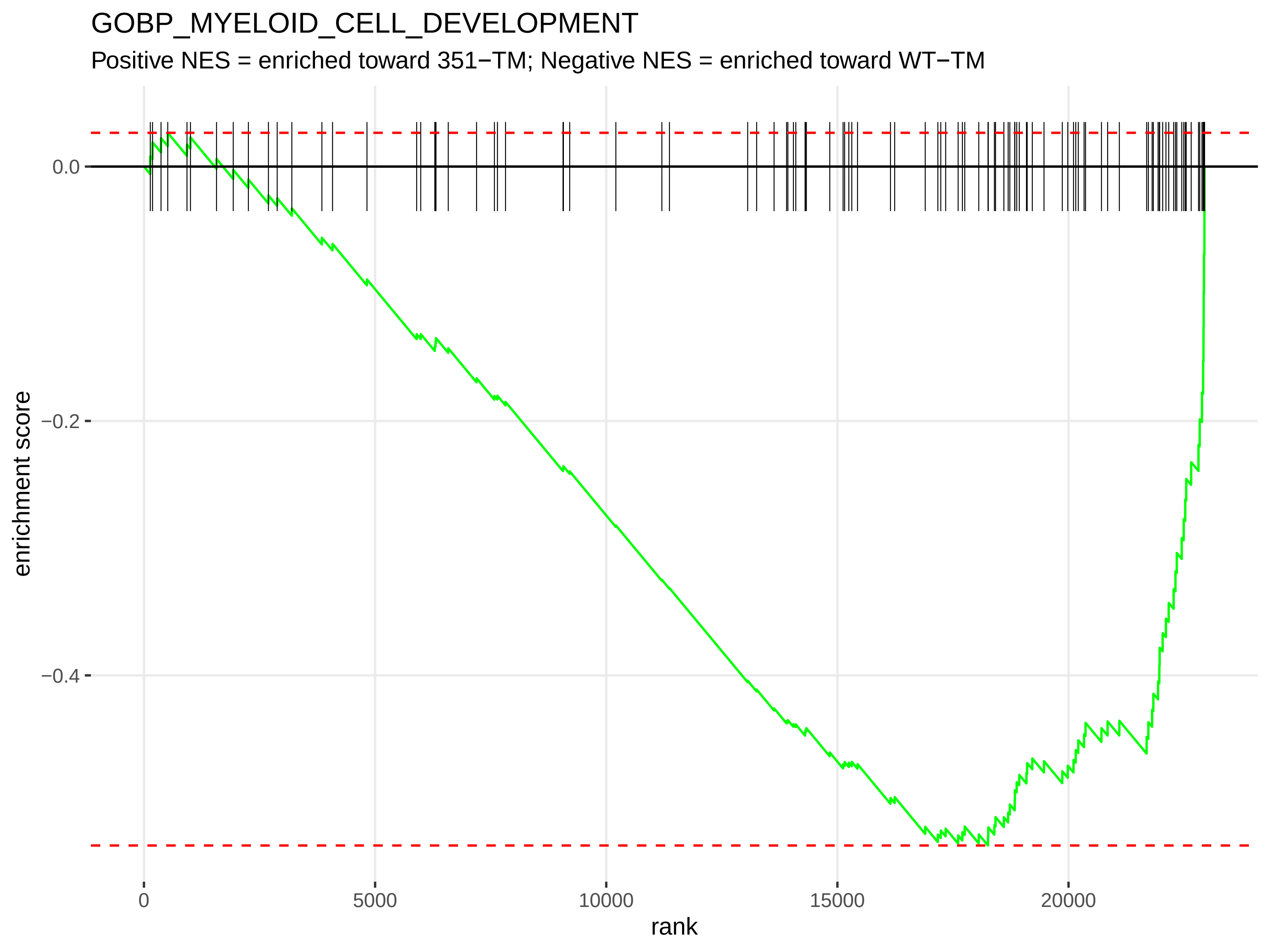

GOBP_MYELOID_CELL_DEVELOPMENT
Positive NES = enriched toward 351−TM; Negative NES = enriched toward WT−TM
enrichment score
0.0
−0.2
−0.4
rank
0
5000
10000
15000
20000

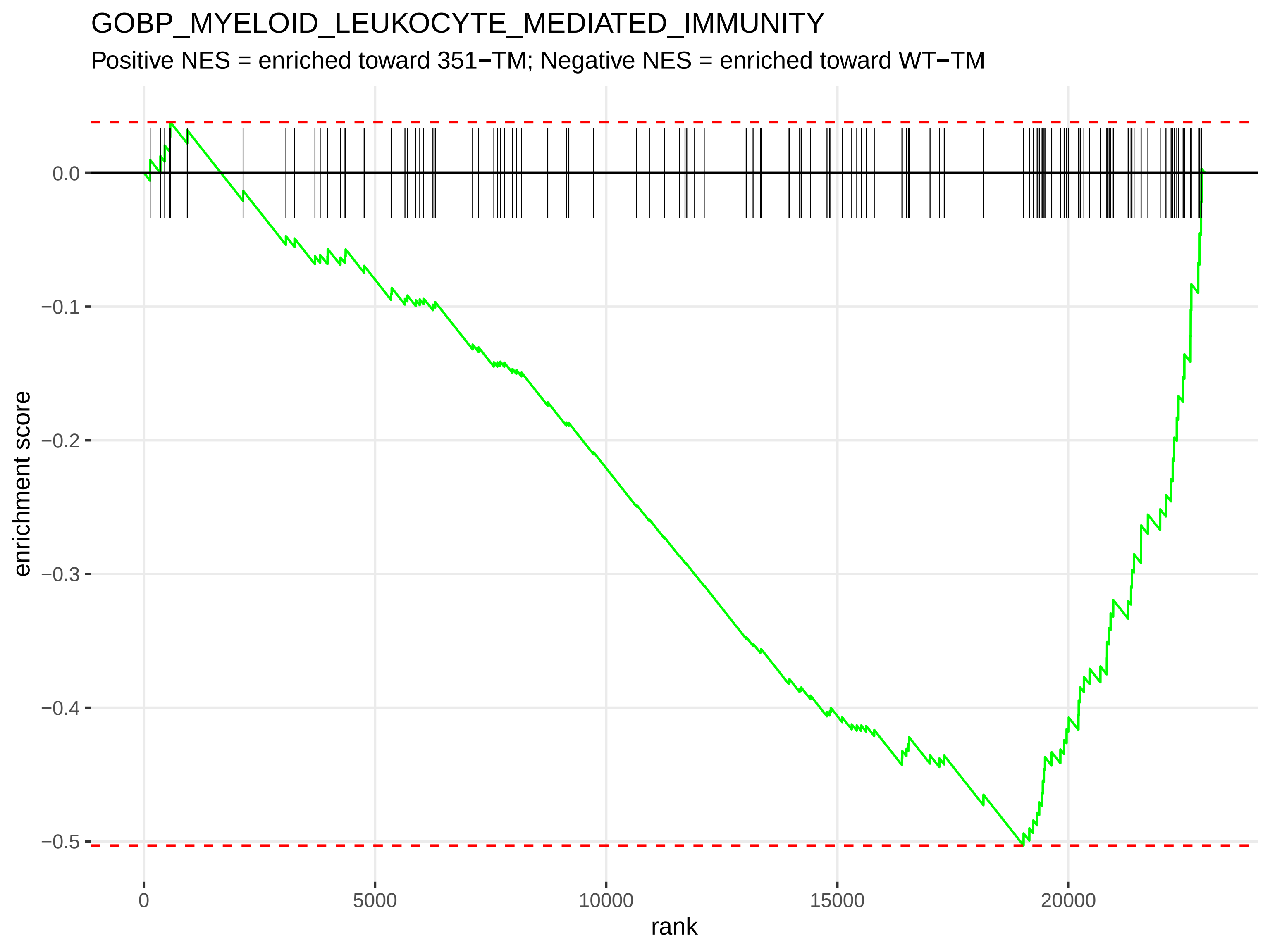
GOBP_MYELOID_LEUKOCYTE_MEDIATED_IMMUNITY
Positive NES = enriched toward 351−TM; Negative NES = enriched toward WT−TM
enrichment score
0.0
−0.1
−0.2
−0.3
−0.4
−0.5
0
5000
10000
15000
20000
rank
